# Carbon Dioxide Separation from Flue Gases: A Technological Review Emphasizing Reduction in Greenhouse Gas Emissions

**DOI:** 10.1155/2014/828131

**Published:** 2014-02-17

**Authors:** Mohammad Songolzadeh, Mansooreh Soleimani, Maryam Takht Ravanchi, Reza Songolzadeh

**Affiliations:** ^1^Department of Chemical Engineering, Amirkabir University of Technology, P.O. Box 15875-4413, Tehran, Iran; ^2^Catalyst Research Group, Petrochemical Research and Technology Company, National Petrochemical Company, P.O. Box 1435884711, Tehran, Iran; ^3^Department of Petroleum Engineering, Petroleum University of Technology, P.O. Box 6198144471, Ahwaz, Iran

## Abstract

Increasing concentrations of greenhouse gases (GHGs) such as CO_2_ in the atmosphere is a global warming. Human activities are a major cause of increased CO_2_ concentration in atmosphere, as in recent decade, two-third of greenhouse effect was caused by human activities. Carbon capture and storage (CCS) is a major strategy that can be used to reduce GHGs emission. There are three methods for CCS: pre-combustion capture, oxy-fuel process, and post-combustion capture. Among them, post-combustion capture is the most important one because it offers flexibility and it can be easily added to the operational units. Various technologies are used for CO_2_ capture, some of them include: absorption, adsorption, cryogenic distillation, and membrane separation. In this paper, various technologies for post-combustion are compared and the best condition for using each technology is identified.

## 1. Introduction

There are ten primary GHGs including water vapor (H_2_O), carbon dioxide (CO_2_), methane (CH_4_), and nitrous oxide (N_2_O) that are naturally occurring. Perfluorocarbons (CF_4_, C_2_F_6_), hydrofluorocarbons (CHF_3_, CF_3_CH_2_F, and CH_3_CHF_2_), and sulfur hexafluoride (SF_6_), are only present in the atmosphere due to industrial processes. Water vapor is the most important, abundant and dominant greenhouse gas, and CO_2_ is the second-most important one (Table 1). Concentration of water vapor depends on temperature and other meteorological conditions, and not directly upon human activities. So it was not indicated in Table 1 [1–3].

**Figure 1:**
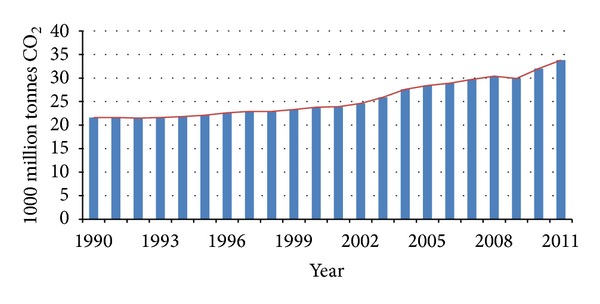
Global CO_2_ emissions from fossil fuel combustion and cement production [23].

**Figure 2:**
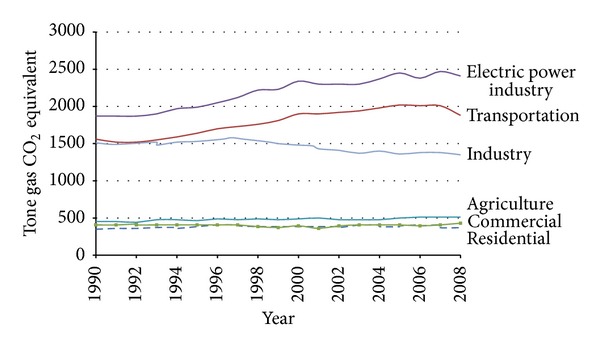
U.S. GHG Emissions Allocated to Economic Sectors [2].

CO_2_ is the primary anthropogenic greenhouse gas, accounting for 77% of the human contribution to the greenhouse effect in recent decade (26 to 30 percent of all CO_2_ emissions). Main anthropogenic emissions of CO_2_ come from the combustion of fossil fuels. CO_2_ concentration in flue gases depends on the fuel such as coal (12–15 mol-% CO_2_) and natural gas (3-4 mol-% CO_2_). In petroleum and other industrial plants, CO_2_ concentration in exhaust stream depends on the process such as oil refining (8-9 mol% CO_2_) and production of cement (14–33 mol-% CO_2_) and iron and steel (20–44 mol-%). From 2004 to 2011, global CO_2_ emissions from energy uses were increased 26% (Figure 1) [4–10].  Figure 2 indicates that power plant (55% of global CO_2_ emissions), transportation (23%), and industry (19%) have highest share in the CO_2_ emission in USA. Cement and petrochemical plants are two major industries for CO_2_ emission, such that cement industry contributes about 5% to global anthropogenic CO_2_ emissions. Also, petrochemical industries are a large share of CO_2_ emission; for example, only in Iran, petrochemical industries emission was about 15 Mton CO_2_/year [11–16].

The Kyoto Protocol is the first international agreement on emissions of GHGs. In this protocol, industrialized countries agreed to stabilize or reduce the GHGs emissions in the commitment period 2008–2012 by 5.2% on average (compared to their 1990 emissions level). Overall, the result of global CO_2_ emissions (Figure 1) shows the failure of Kyoto protocol; therefore, in 2011 Durban COP meeting, this protocol was extended until 2017. Several countries with high GHGs emission like China, India, Brazil, and even Iran have added to this Protocol. Intergovernmental Panel on Climate Change (IPCC) predicted the atmosphere may contain up to 570 ppmv CO_2_ by the year 2100, causing a rise of mean global temperature and sea level around 1.9°C and 38 m, respectively [15, 17–20]. Given that the earth's average temperature continues to rise, Intergovernmental Panel on Climate Change (IPCC) stated, global GHG emissions must be reduced by 50 to 80 percent by 2050 to avoid dramatic consequences of global warming [21–23].

Carbon capture and storage (CCS) is the most indicated technology to decrease CO_2_ emission from fossil fuels sources to atmosphere. Also, CO_2_ separated from flue gases can be used in enhanced oil recovery (EOR) operations where CO_2_ is injected into oil reservoirs to increase mobility of oil and reservoir recovery [24, 25]. Pure CO_2_ has many applications in food/beverage and different chemical industries such as urea and fertilizer production, foam blowing, carbonation of beverages and dry ice production, or even in the supercritical state as supercritical solvent [26–28].

From this definition, CCS consists of three basic stages: (a) separation of CO_2_, (b) transportation, and (c) storage. Operating costs of these stages have been estimated in 2008:

- CO_2_ separation from exhausting gases: 24 to 52 €/ton-CO_2_,
- transportation to storage location: 1 to 6 €/ton-CO_2_ per 100 km,
- storage: −28 to 42 €/ton-CO_2_.

**Figure 3:**
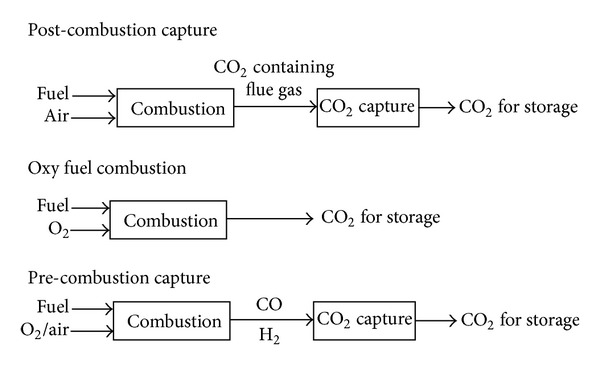
Three basic approaches of CO_2_ capture [29].

Therefore, CO_2_ separation is a major stage in CCS. The CCS total costs can vary from −3 to 106 €/ton-CO_2_ (negative values are expected for the injection of CO_2_ in EOR). There are three major approaches for CCS: pre-combustion capture, oxy-fuel process, and post-combustion capture (Figure 3) [25, 30, 31].

Pre-combustion capture involves reaction of a fuel with oxygen or air and in some cases steam to produce a gas mainly composed of carbon monoxide and hydrogen, which is known as synthesis gas (syngas) or fuel gas. The produced carbon monoxide is reacted with steam in a catalytic reactor, called shift converter, to give CO_2_ and more hydrogen. CO_2_ is then separated, usually by cryogenic distillation or chemical absorption process, resulting in a hydrogen-rich fuel that can be used in many applications, such as furnaces, gas turbines, engines and fuel cells [32, 33].

A main advantage of post-combustion is the higher CO_2_ concentration and pressure achieved in the output stream. The main disadvantage of pre-combustion capture is system needs long-term development in a number of enabling technical areas to achieve targeted efficiency towards a hydrogen economy. This disadvantage has limited application of this approach and increased investments costs of pre-combustion capture [34, 35].

In oxy-fuel combustion, nearly pure oxygen is used for combustion instead of ambient air, and this results in a flue gas that is mainly CO_2_ and H_2_O, which are separated by condensing water. Three major advantages of this method are high CO_2_ concentration in output stream (above 80% v/v), high flame temperature, and easy separation of exhaust gases. The major disadvantages of oxy-fuel combustion are high capital cost and large electric power requirement to separate oxygen from air [36–38].

The principle of post-combustion capture is CO_2_ separation from flue gas after combustion. Generally, the CO_2_ in flue gas is diluted (8–15% CO_2_) with inert gases such as nitrogen, argon, and water in addition to oxygen. Flue gases are normally at atmospheric pressure and high temperatures (between 320 K and 400 K) [39–41]. Post-combustion capture does not require expensive technologies such as syngas separation, hydrogen turbine, fuel cell. Post-combustion capture is the most important to prevent CO_2_ emissions, because it offers flexibility and does not need to change combustion cycle. If the capture plant shuts down, the power plant can still operate [42, 43]. Major disadvantage of this method is unfavorable condition of flue gases.

Because of the importance in selecting suitable process for CO_2_ separation, in this research various technologies for this purpose have been focused.

## 2. CO_**2**_ Separation Technologies

**Figure 4:**
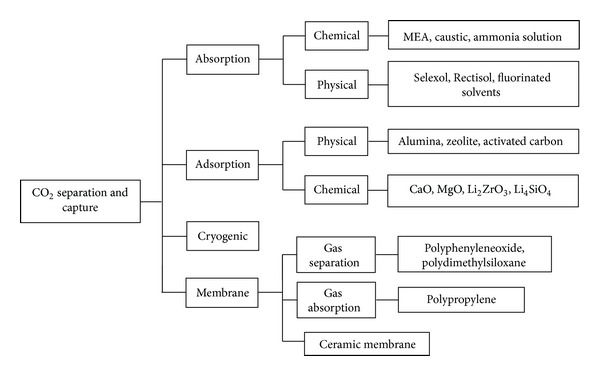
Different technologies for CO_2_ separation [29].

Based on economical and environmental considerations, it is necessary to apply efficient and suitable technology for CO_2_ separation with low operating cost and energy consumption. Up to now, there are several gas separation technologies being investigated for post-combustion capture, namely, (a) absorption, (b) adsorption, (c) cryogenic distillation, and (d) membrane separation (Figure 4) [39, 44]. Although various new methods were suggested for CO_2_ separation, Granite and Brien [45] reviewed some of the most novel methods for carbon dioxide separation from flue and fuel gas streams, such as use of electrochemical pumps and chemical looping for CO_2_ separation.

### 2.1. Absorption

**Figure 5:**
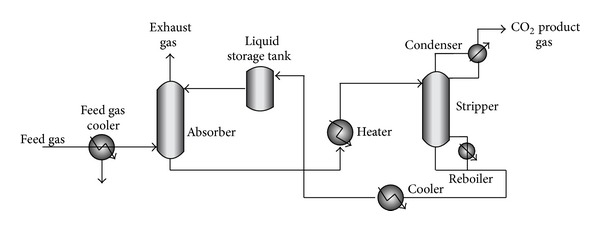
Schematic diagram of CO_2_ absorption pilot plant.

Absorption stripping is an important technology for CO_2_ capture from fuel gas; in this technology desired component in mixed gases are dissolved in a solvent (bulk phase) [46]. The general scheme of this process is depicted in Figure 5.

The flue gas (containing CO_2_) is cooled (between 318 and 323 K), and fed to the absorption column (scrubber) where the solvent absorbs CO_2_. The CO_2_-rich solution is fed into a heater to increase the temperature of solution, then to a stripper column to release the CO_2_. The released CO_2_ is compressed, and the regenerated absorbent solution is cooled and recycled to the absorber column [47, 48].

Energy required for post-combustion CO_2_ capture is an important issue. Thus, recent studies suggest that reduction of the cost of this capture could be achieved by finding suitable solvents that could process larger amounts of CO_2_ for a given mass and require less energy for stripping stage [49, 50].

#### 2.1.1. Solvents

In absorption process, flue gas is contacted with a liquid “absorbent” (or “solvent”), and CO_2_ is absorbed by this solvent [21]. However, the absorbent should have a suitable capacity for CO_2_ absorption, high kinetic rate for CO_2_ absorption, negligible vapor pressure, and high chemical and thermal stability and should be harmless for labor persons [51–53].

The solvents used for CO_2_ absorption can be divided into two categories: physical and chemical solvents. Physical solvent processes use organic solvents to physically absorb acid gas components rather than reacting chemically, but chemical absorption depends on acid-base neutralization reactions using alkaline solvents [54, 55]. In the recent years, many studies have compared the performance of different solvents as listed in Table 2.

*(1) Alkanolamines.* Between various solvent groups, alkanolamines group is the most important and more used for CO_2_ separation. A major problem in the usage of amines for CO_2_ absorption is equipment corrosion, so Albritton et al. [56] examined corrosion rate of various amine solvents and suggested corrosion rate could reduce in the following order: monoethanolamine (MEA) > 2-amino-2-methyl-1-propanol (AMP) > diethanolamine (DEA) > methyl diethanolamine (MDEA).

On the other way, MEA can react more quickly with CO_2_ than MDEA, but MDEA has higher CO_2_ absorption capacity and requires lower energy to regenerate CO_2_ [39, 57, 58]. Thus, it can be concluded that MEA is one of the best amine solvents for CO_2_ separation. Idem et al. [59] reported substantial reduction in energy requirements and modest reduction in circulation rates for amine blends relative to the corresponding single amine system of similar total amine concentration. Wang et al. [57] found that when MEA and MDEA are mixed at the appropriate ratio, the energy consumption for CO_2_ regeneration is reduced significantly. Dave et al. [28] compared the performance of several amine solvents and ammonia solutions at various concentrations. They showed that 30 wt% AMP based process has the lowest overall energy requirement among the solvents considered in their study (30% MEA, 30% MDEA, 2.5% NH_3_, and 5% NH_3_) [28, 60].

Knudsen et al. [61] studies showed that it is possible to run the post-combustion capture plant continuously while achieving roughly 90% CO_2_ separation levels and CASTOR-2 (blended amine solvents), operated in pilot scale with lower steam requirement and liquid-to-gas ratio (L/G) than the conventional MEA solvent.

Besides alkanolamines, carbonate-bicarbonate buffers and hindered amines are used in the bulk removal of CO_2_ owing to the low steam requirement for its regeneration. Mitsubishi Heavy Industries and Kansai Electric have employed other patented chemical solvents—strictly hindered amines called KS-1, KS-2, or KS-3. The regeneration heat of KS solvents is said to be ~3 GJ/t CO_2_, that is, 20% lower than that of MEA with ~3.7 GJ/t CO_2_ [60, 64, 77]. Generally, the overall cost of amine absorption/stripping technology for CO_2_ capture process is 52–77 US$/ton CO_2_ [71].

*(2) Amino Acid.* Amino acids have the same functional groups as alkanolamines and can be expected to behave similarly towards CO_2_ but do not deteriorate in the presence of oxygen. Based on the results of tests, the aqueous potassium salts (composed of sarcosine and proline) are considered to be the most promising solvents. The most common amino acids used in the gas treating solvents are glycine, alanine, dimethyl glycine, diethyl glycine, and a number of sterically hindered amino acids [65, 67, 68].

Results of many research groups showed that these solvents are suitable for application in membrane gas absorption units, because these solvents have better performance and degradation resistance than other chemical solvents [78]. Amino acid salts formed by neutralization of amino acids with an organic base such as amine showed better CO_2_ absorption potential than amino acid salts from neutralization of amino acid salts from an inorganic base such as potassium hydroxide [79, 80]. Aronu et al. [69] studied the performance of amino acids neutralized with 3-(methylamino)propylamine (MAPA), glycine, *β*-alanine, and sarcosine. Their results indicated that sarcosine neutralized with MAPA has the best CO_2_ absorption performance. Its performance is also enhanced by promoting with excess MAPA [69].

**Figure 6:**
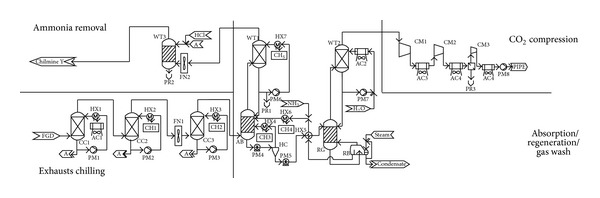
Schematic layout of CO_2_ separation block based on the chilled ammonia process [92].

*(3) Ammonia.* Since ammonia is a toxic gas, prevention of ammonia “slip” to the atmosphere is a necessity. Despite this disadvantage, chilled ammonia process (CAP) was used for CO_2_ separation (Figure 6). In the CAP, CO_2_ is absorbed in an ammoniated solution at a lower absorption temperature (275–283 K) that reduced ammonia emissions from the CAP absorber. Ammonium carbonate solution resulted in approximately 38% carbon regeneration compared to MEA solution [70, 81, 82].

*(4) Aqueous Piperazine (PZ)*. Piperazine (PZ) is as an additive used for amine systems to improve kinetics of CO_2_ absorption, such as MDEA/PZ or MEA/PZ blends. Because PZ solubility in water is low, concentration of PZ is between 0.5 and 2.5 M. As indicated in Table 2, increasing the concentration of PZ in solution allows for increased solvent capacity and faster kinetic. The presence of potassium in solution increases the concentration of CO_3_^2−^/HCO_3_^−^ in solution; therefore, solution has buffering property. These competing effects yield a maximum fraction of reactive species at potassium to piperazine ratio of 2 : 1 [75, 83, 84].

### 2.2. Adsorption

Adsorption operation can reduce energy and cost of the capture or separation of CO_2_ in post-combustion capture. To achieve this goal, it is necessary to find adsorbents with suitable properties. In general, CO_2_ adsorbent must have high selectivity and adsorption capacity and adequate adsorption/desorption kinetics, remain stable after several adsorption/desorption cycles, and possess good thermal and mechanical stability [51, 85–88]. The adsorbents used for CO_2_ separation placed into two main categories: physical and chemical adsorbents.

#### 2.2.1. Chemical Adsorption

Chemisorption is a subclass of adsorption, driven by a chemical reaction occurring at the exposed surface. Adsorption capacities of different chemical adsorbents are summarized in Table 3.

A wide range of metals have been studied including [89]

- metal oxides: CaO, MgO,
- metal salts from alkali metal compounds: lithium silicate, lithium zirconate to alkaline earth metal compounds (i.e., magnesium oxide and calcium oxide),
- hydrotalcites and double salts.

In general, one mole of metal compound can react with one mole of CO_2_ with a reversible reaction. The process consists of a series of cycles where metal oxides (such as CaO) at 923 K are transformed into metal carbonates form (such as CaCO_3_) at 1123 K in a carbonation reactor to regenerate the sorbent and produce a concentrated stream of CO_2_ suitable for storage [90, 91].

Considerable attention was paid to calcium oxide (CaO) as it has a high CO_2_ adsorption capacity and high raw material availability (e.g., limestone) at a low cost. Lithium salts was recorded a good performance in CO_2_ adsorption, but it gained less focus due to its high production cost. Although double salts can be easily regenerated due to low energy requirement, their stability has not been investigated [93, 96].

The reaction of CO_2_ adsorption with Li_2_ZrO_3_ is reversible in the temperature range of 723–863 K. The capacity of lithium silicate (8.2 moL CO_2_/kg sorbent at 993 K) is larger than that of lithium zirconate (4.85 moL/kg sorbent) [17].

Hydrotalcite (HT) contains layered structure with positively charged cations balanced by negatively charged anions [97, 98]. Adsorption and final capacity of different adsorption/desorption cycles are listed in Table 3.

One way for improving CO_2_ adsorption efficiency is application of nanomaterials. Different nano-materials can be used for CO_2_ separation (Table 3). However, nanomaterials always have high production cost with complicated synthesis process such as carbon nanotubes and graphite nanoplatelets [99, 100].

The main disadvantage of chemical adsorbents is difficult regeneration process, and application of these adsorbents needs more studies for finding new adsorbents [88, 95].

#### 2.2.2. Physical Adsorption

Physisorption, also called physical adsorption, is a process in which the electronic structure of the atom or molecule is barely perturbed upon adsorption. If the CO_2_ adsorption capacity of solid adsorbents reaches 3 mmoL/g, the required energy for adsorption will be less than 30–50% energy for absorption with optimum aqueous MEA [101]. The major physical adsorbents suggested for CO_2_ adsorption include activated carbons and inorganic porous materials such as zeolites [102, 103]. The adsorption capacities of various physical adsorbents are summarized in Table 4.

Coal is one of the adsorbents being suggested for CO_2_ separation. The total amount of CO_2_ that can be adsorbed in coal depends on its porosity, ash, and affinity for this molecule [111, 112]. Sakurovs et al. [113] showed that the ratio of maximum sorption capacity between CO_2_ and methane decreases with increasing carbon content. The average CO_2_/CH_4_ sorption ratio is higher for moisture-equilibrated coal and decreases with increasing coal rank (1.4 for high rank coals to 2.2 for low rank coals) [114–116].

Activated carbon (AC) has a number of attractive characteristics, such as its high adsorption capacity, high hydrophobicity, low cost, and low energy requirement for regeneration [117–119]. Activated carbons are inexpensive, insensitive to moisture, and easy for regeneration. These adsorbents have well developed micro- and mesopore structures that are suitable for high CO_2_ adsorption capacity at ambient pressure [120–122].

However, activated carbon CO_2_/N_2_ selectivities (ca. 10) are relatively low; zeolitic materials offer CO_2_/N_2_ selectivities 5–10 times greater than those of carbonaceous materials. The adsorption capacity and selectivity of zeolites are largely affected by their size, porous diameter, charge density, and chemical composition of cations in their porous structures. The average value of heat adsorption on zeolites (36 kJ/moL) is larger than for activated carbon (30 kJ/moL), confirming the mentioned affirmation. Moreover, activated carbon can be regenerated easily and completely. Also its capacity did not decay after 10 consecutive processes cycles [122–124].

Due to the increase in cost of raw materials, growing research interest has been focused on producing AC from agricultural wastes. Some of the agricultural wastes include the shells and stones of fruits, wastes resulting from the production of cereals, bagasse, and coir pith [100]. Rosas et al. [125] prepared hemp-derived AC monolith by phosphoric acid activation. The activated carbons from hemp stem are microporous materials and therefore suitable ones for hydrogen storage and CO_2_ capture [126].

Siriwardane et al. [127] studied CO_2_ adsorption on the molecular sieve 13X, 4A and activated carbon. The molecular sieve 13X showed better CO_2_ separation than molecular sieve 4A. At lower pressures (<50 psi), activated carbon had a lower CO_2_ separation than the molecular sieves, but adsorption was higher for activated carbon than molecular sieves at higher pressures [127, 128].

Liu et al. [129] indicated that zeolite 5A has higher volumetric capacities and less severe heat effect of the zeolite 13X. Chabazite zeolites were prepared and exchanged with alkali cations: Li, Na, K and alkaline-earth cations: Mg, Ca, Ba. Zhang et al. [130] studied the potential of these zeolites for CO_2_ separation from flue gas by vacuum swing adsorption. It was found that NaCHA and CaCHA hold comparative advantages for high temperature CO_2_ separation whilst NaX showed superior performance at relatively low temperatures [130]. In physical adsorption, the size and volume of the pores are important. Micropores are defined as pores, 2 nm in size, mesopores between 2 and 50 nm, and macropores, 50 nm in size. The micropores make better selective adsorption of CO_2_ over CH_4_ [131, 132].

Carbon nanotubes (CNTs) are the most famous among nano-hollow structured materials and their dimension ranges from 1 to 10 nm in diameter and from 200 to 500 nm in length [133]. Cinke et al. [134] indicated that purified single-walled carbon nanotubes (SWNTs) adsorbed CO_2_ better than unpurified SWNT. In addition, multiwalled carbon nanotubes (MWNTs) showed stability for 20 cycles of adsorption and regeneration [135].

More recently, nanosystems researchers have synthesized and screened a large number of zeolitic-type materials known as zeolitic imidazolate frameworks (ZIFs). CO_2_ capacities of the ZIFs are high, and selectivity against CO and N_2_ is good [136, 137]. The results of researchers (Burchell and Judkins [138], Dave et al. [28], and Yong et al. [139]) indicated that the CO_2_ adsorption efficiency of the honeycomb monolith is twice than activated carbon and 1.5 times greater than ZIF material [29]. Results of Kimber et al. [140] showed that CO_2_ selectivity of honeycomb monolithic composite decreased with increasing in burn-off.

Graphite nanoplatelets (GNP) were prepared by acid intercalation followed by thermal exfoliation of natural graphite. Functionalized graphite nanoplatelets (f-GNP) were prepared by further treatment of GNP in acidic medium. Palladium (Pd) nanoparticles were decorated over f-GNP surface by chemical method [109, 141, 142]. Adsorption capacity of this adsorbent is presented in Table 4.

The presence of several impurity gases (SO_*x*_/NO_*x*_/H_2_O) greatly complicates the CO_2_ separation processes. Therefore, conventional adsorption-based CO_2_ separation processes rely on using a pretreatment stage to remove water, SO_*x*_, and NO_*x*_, which adds considerably to the overall cost. Also this prelayer can be used before the amine absorption column [143, 144]. Deng et al. [145] showed that the adsorption capacities follows the order SO_2_ > CO_2_ > NO > N_2_ on both zeolites (5A and 13X). Comparing two different adsorbents, the better separation efficiency can be achieved by 5A zeolite [145].

Zhang et al. [130] focused on the effect of water vapour on the pressure/vacuum swing adsorption process. The selected adsorbents in this study were CDX (an alumina/zeolite blend), alumina, and 13X zeolite as these adsorbents are either the prelayer for water adsorption or the main CO_2_ adsorption layer in the packed bed [130].

Metal-organic framework (MOF) materials are crystalline with two- or three-dimensional porous structures that can be synthesised with many of the functional capabilities of zeolites. Several MOFs have been proposed as adsorbents for CO_2_ separation processes, and among these Cu-BTC [polymeric copper (II) benzene-1,3,5-tricarboxylate] has proved to be dedicated with CO_2_ adsorption performances that are higher than those of typical adsorbents such as 13X zeolite [105, 107, 146, 147].

The MCM-41 material is one of the mesoporous products which was prepared by the hydrothermal method from mobil composition of matter (MCM) powders. Lu et al. [148] showed that mesoporous silica spherical particles (MSPs) can be synthesized using low-cost Na_2_SiO_3_ thus they can be cost-effective adsorbents for CO_2_ separation from flue gas [149, 150].

Layered double hydroxides (LDHs) have general formula [M_1−*x*_^II^M_*x*_^III^(OH)_2_][X_*x*/C_^*g*−^ · *n*H_2_O] with *x* typically in the range between 0.10 and 0.33. These materials can be readily and inexpensively synthesized with the desired characteristics for a particular application such as CO_2_ adsorption [108, 151].

#### 2.2.3. Adsorbent Modification

The role of CO_2_ as a weak Lewis acid is well established. Because of the nature of CO_2_, the surface of the physical adsorbents can be modified by adding basic groups, such as amine groups and metal oxides to improve CO_2_ adsorption capacity or selectivity [152–154]. Three different methods for the production of these adsorbents were investigated: activation with CO_2_, heat treatment with ammonia gas (amination and ammoxidation), and heat treatment with polyethylenimine (PEI). However, it has been suggested that amine modification can produce better and cheaper CO_2_ adsorbents [24, 104, 155, 156].

Xu et al. [157, 158] designed selective “molecular basket” by grafting polyethylenimine (PEI) uniformly on MCM-41. CO_2_ adsorption capacity of the adsorbent was 24 times higher than MCM-41 and 2 times higher than PEI [93]. The addition of ammonium hydroxide resulted in the Zr-MOF with a slight lower adsorption of CO_2_ and CH_4_; however, the selectivity of CO_2_/CH_4_ is significantly enhanced [159, 160]. Results of Abid et al. [107] showed that the selectivity of CO_2_/CH_4_ on Zr-MOF is between 2.2 and 3.8, while for Zr-MOF-NH_4_ selectivity is between 2.6 and 4.3.

A nitrogen-rich carbon with a hierarchical micro-mesopore structure exhibited a high CO_2_ adsorption capacity (141 mg/g at 298 K, 1 atm), excellent separation efficiency (CO_2_/N_2_ selectivity is ca. 32), and excellent stability [161]. Plaza et al. [162] results showed that CO_2_ adsorption capacity of the DETA-impregnated alumina (≥2.3 mmoL/g) exhibited is the highest.

Amine modified layered double hydroxides (LDHs) have been prepared by several different methods. Park et al. [163] used dodecyl sulfate (DS) intercalated LDH as precursor and added (3-aminopropyl) triethoxysilane (APTS) together with N-cetyl-N,N,N-trimethylammonium bromide (CTAB) [164]. The highest adsorption capacity of amine modified LDHs for CO_2_ was achieved at 1.75 mmoL/g by MgAl N3 at 353 K and 1 bar. According to data in Table 4, this adsorbent has high CO_2_ capacity at high temperature; therefore, this adsorbent is suitable for post-combustion CO_2_ capture [108].

Wang et al. [114] reported that porous carbons with well-developed pore structures were directly prepared from a weak acid cation exchange resin (CER) by the carbonization of a mixture with Mg acetate in different ratios [108]. The main parameters of this adsorbent (such as CO_2_ capacity) are indicated in Table 4.

Shafeeyan et al. [165] prepared different adsorbents based on the central composite design (CCD) with three independent variables (i.e., amination temperature, amination time, and the use of preheat treated (HTA) or preoxidized (OXA) sorbent as the starting material). They demonstrated that the optimum condition for obtaining an efficient CO_2_ adsorbent is using a preoxidized sorbent and amination at 698 K for 2.1 h [165].

Table 4 compares CO_2_ adsorption capacities and stability of different absorbents, which were studied for post-combustion CO_2_ capture.

#### 2.2.4. Different Cycles for CO_**2**_ Adsorption

**Figure 7:**
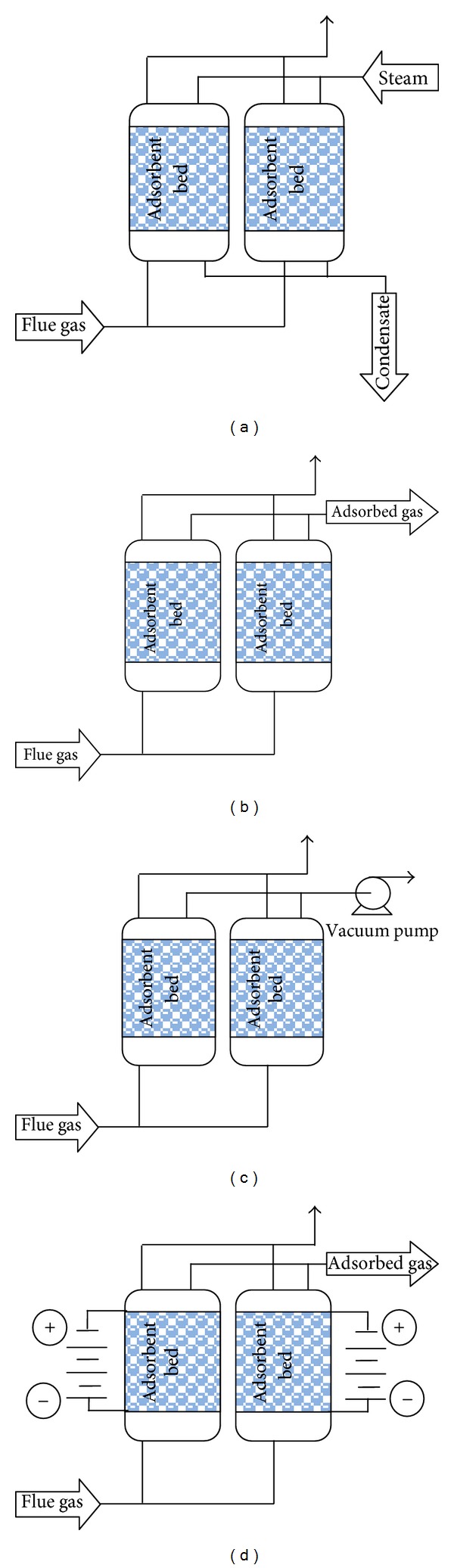
Schematic diagrams of various adsorption cycles, (a) TSA, (b) PSA, (c) VSA, and (d) ESA; thin lines indicated operation streams in regenerated step.

Five different regeneration strategies were demonstrated in a single-bed CO_2_ adsorption unit: pressure swing adsorption (PSA), temperature swing adsorption (TSA), vacuum swing adsorption (VSA), electric swing adsorption (ESA), and a combination of vacuum and temperature swing adsorption (VTSA). The difference between these technologies is based on the strategy for regeneration of adsorbent after the adsorption step (Figure 7). In PSA applications, the pressure of the bed is reduced. VSA is preferred to the special PSA application where the desorption pressure is below atmospheric, whereas in TSA, the temperature is raised while pressure is maintained approximately constant, and in ESA the solid is heated by the Joule effect [166–169].

For the single-bed cycle configurations, the productivity and CO_2_ recovery followed the sequence:

(1) $$\begin{array}{c} \text{ESA}<\text{TSA}<\text{PSA}<\text{VSA}<\text{VTSA}. \end{array}$$

The performances of PSA, TSA, VSA, VTSA, and ESA processes for CO_2_ separation are reported in Table 5. Since application of adsorption process for CO_2_ capture in industrial scale is very important, in recent years some researches have been focused on this area; for example, Lucas et al. [170] studied the scale-up CO_2_ adsorption with activated carbon.

### 2.3. Cryogenic Distillation

Cryogenic method utilized low temperatures for condensation, separation, and purification of CO_2_ from flue gases (freezing point of pure CO_2_ is 195.5 K at atmospheric pressure). Therefore, under the cryogenic separation process, the components can be separated by a series of compression, cooling, and expansion steps. It enables direct production of liquid CO_2_ that can be stored or sequestered at high pressure via liquid pumping [171–173].

The advantages of this technology can be summarized as follows [6, 8, 174].

1. Liquid CO_2_ is directly produced, thus making it relatively easy to store or send for enhanced oil recovery.
2. This technology is relatively straightforward, involving no solvents or other components.
3. The cryogenic separation can be easy scaled-up to industrial-scale utilization.

The major disadvantages of this process are the large amount of energy required to provide the refrigeration and the CO_2_ solidification under a low temperature, which causes several operational problems [176–178]. Therefore, more studies are required for reducing the cost of cryogenic separation.

Clodic et al. [179] indicated that the energy requirement for cryogenic process was in the range of 541–1119 kJ/kg CO_2_. Zanganeh et al. [6] have constructed a pilot-scale CO_2_ capture and compression unit (CO_2_ CCU) that can separate CO_2_ as liquid phase from the flue gas of oxy-fuel combustion. Their results showed that cryogenic is the most cost effective when the feed gas is available at high pressure. Therefore, cryogenic is not suitable for post-combustion and it is well effective for separation stream with high CO_2_ concentration such as oxy-fuel combustion. Amann et al. [180] reported that conversion of O_2_/CO_2_ cycle was more efficient than amine scrubbing but more difficult to implement because of the specific gas turbine.

**Figure 8:**
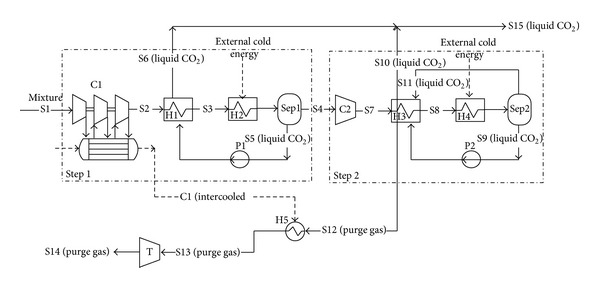
Novel CO_2_ cryogenic liquefaction and separation system [175].

Xu et al. [175] studied a novel CO_2_ cryogenic liquefaction and separation system (Figure 8). In this system, two-stage compression, two-stage refrigeration, two-stage separation, and sufficient recovery of cryogenic energy were adopted. The energy consumption for CO_2_ recovery is only 0.395 MJ/kg CO_2_. Furthermore, this CO_2_ cryogenic separation system is more suitable for gas mixtures with high initial pressure and high CO_2_ concentration [175].

Song et al. [181] developed a novel cryogenic CO_2_ capture system based on Stirling coolers (SC). The operation of Stirling cooler contains four processes: isothermal expansion, refrigeration under a constant volume, isothermal compression, and heating under a constant volume condition. This novel cryogenic system can condense and separate H_2_O and CO_2_ from flue gas. Their results showed that under the optimal temperature and flow rate, CO_2_ recovery of the cryogenic process can reach 96% with 1.5 MJ/kg CO_2_ energy consumption.

**Figure 9:**
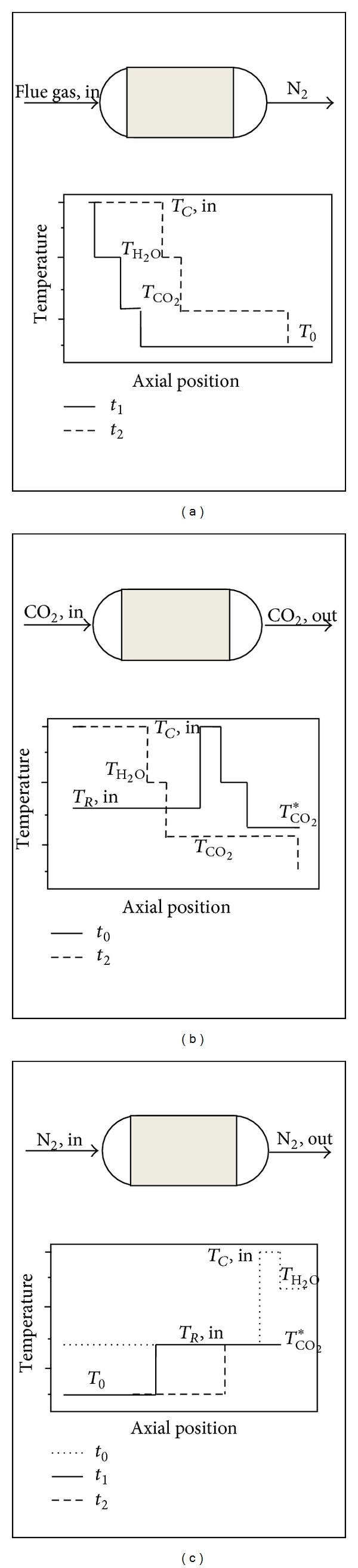
Schematic axial temperature and corresponding mass deposition profiles for the cryogenic; (a) capture, (b) recovery, and (c) cooling cycles [182].

Tuinier et al. [182] exploited a novel cryogenic CO_2_ capture process using dynamically operated packed beds (Figure 9). By the developed process, above 99% of CO_2_ could be recovered from a flue gas containing 10 vol.% CO_2_ and 1 vol.% H_2_O with 1.8 MJ/kg CO_2_ energy consumption [181].

**Figure 10:**
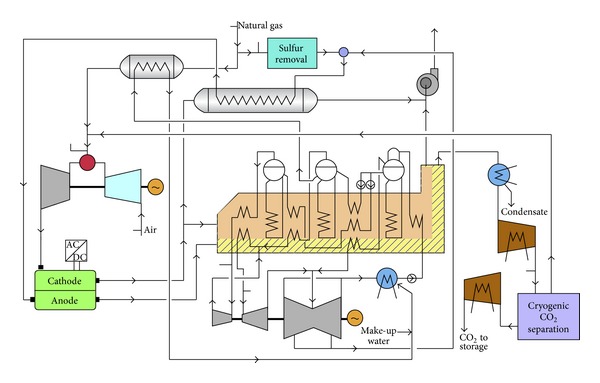
Plant layout showing the integration of the MCFC in a combined cycle, with cryogenic CO_2_ separation after oxygen combustion of the cell an anode exhaust [183].

Chiesa et al. [183] proposed an advanced cycle that a molten carbonate fuel cell (MCFC) was used to separate the CO_2_ from the gas turbine exhaust of a natural gas fired combined cycle power plant. In this cycle, gas turbine flue gases actually are used as cathode feeding for MCFC. While CO_2_ is moved from the cathode to anode side, concentrate CO_2_ in the anode exhaust. Then the CO_2_ is concentrated on the anode side of MCFC allowing to easily treat this spent fuel stream in a cryogenic process to split combustible species (routed back to gas turbine combustor) from the CO_2_ addressed to storage (Figure 10) [183].

### 2.4. Membrane Separation

The membrane separation method is a continuous, steady-state, clean and simple process, and ideal as an energy-saving method for CO_2_ recovery. Gas separation using membranes is a pressure-driven process. Due to the low pressure of flue gases, driving force is too low for membrane processes in post-combustion (low pressure and low CO_2_ concentration). Membrane processes offer increased separation performances when CO_2_ concentration in the feed mixture increases [184–186].

Membrane separation processes have several advantages over other CO_2_ separation technologies. The required process equipment is very simple, compact, relatively easy to operate and control, clear process and easy to scale up [187, 188].

The energy required for the recovery of CO_2_ by membrane processes depends on the target purity, flue gas composition, and membrane selectivity for CO_2_. Howevre membrane processes require too much energy for post-combustion CO_2_ capture; therefore, low partial pressure of CO_2_ in the flue gas is a possible disadvantage for the application of membranes. Another disadvantage of membrane process is that the membrane selectivity for the separation of CO_2_ from SO_*x*_ and NO_*x*_ is very low. Membrane process is not useful for high flow rate applications [189–191].

Therefore, the useful membrane for post-combustion CO_2_ capture should have some specification such as [192, 193]

- high CO_2_ permeability,
- high selectivity for CO_2_ separatation from flue gases,
- high thermal and chemical stability,
- resistant to plasticisation,
- resistant to aging,
- cost effective,
- low production cost for different membrane modules.

Many efforts have been made to find new material with suitable properties (Table 6).

Various groups of materials have been already proposed and experimentally investigated for post-combustion CO_2_ capture with membrane process. By modifying membrane their properties can be improved. For example, when amine functional groups are randomly dispersed in the silica matrix, this membrane can separate CO_2_ with high selectivity. On the other hand, membrane structure can be modified by adding arginine salts [194–196].

#### 2.4.1. Inorganic Membranes

Based on structure, inorganic membranes can be classified into two categories: porous and dense. In porous inorganic membranes, a porous thin top layer is supported on a porous metal or ceramic support. Zeolite, silicon carbide, carbon, glass, zirconia, titania, and alumina membranes are mainly used as porous inorganic membranes supported on different substrates, such as *α*-alumina, *γ*-alumina, zirconia, zeolite, or porous stainless steel [17, 199, 209, 210].

Zeolite membrane is the most important group of inorganic membranes. Zeolite membranes are considered more expensive than polymeric membranes, and therefore their unique properties of size selectivity and thermal and chemical stability should be exploited for successful application [211–213].

The dense inorganic membranes (nonporous material) consist of a thin layer of metal, such as palladium and its alloys (metallic membrane), or solid electrolytes, such as zirconia. Another form of inorganic membrane is the liquid-immobilized membrane, where the pores of a membrane are completely filled with a liquid, which is permselective for certain compounds. Recently, attempts have been made to develop dense molten carbonate selective membranes for CO_2_ separation at high temperatures (>723 K). The inorganic membranes have high thermal stability for CO_2_ separation, but their selectivity and permeability are very low [200, 214, 215].

#### 2.4.2. Polymeric Membranes

In polymeric membranes, the selective layer is generally a nonporous film that transports gases across by the solution-diffusion mechanism. Polyacetylenes, polyaniline, polyarylene ethers, polyarylates, polycarbonates, polyetherimides, polyethylene oxide, polyimides, polyphenylene oxides, polypyrroles, polysulfones, and amino groups such as polyethyleneimine blends, polymethacrylates are examples of polymeric membranes used for CO_2_ separation [17, 216–218].

Selective polymeric membranes can be divided into two basic categories: glassy and rubbery. Almost glassy polymeric membranes are more suitable than rubbery polymeric membranes for CO_2_ separation because of their high gas selectivity and good mechanical properties. On the other hand, rubbery membranes are flexible and soft and they have a high permeability but a low selectivity, whereas glassy polymers exhibit a low permeability but a high selectivity [206, 219–221].

Several advantages of polymeric membranes are (i) low cost of production; (ii) high performance separation; (iii) ease of synthesis; and (iv) mechanical stability. Although the polymeric membranes have high selectivity and permeability for CO_2_ separation, but their thermal stability is very low, and these membrane may be plasticized with influence of CO_2_ in membrane. Therefore, application of these membranes for post-combustion capture is limited, and flue gas must first be cooled down to 313–333 K for membrane process [184, 222, 223].

Ren et al. [205] prepared polymeric membranes with block copolymers; the balance of the hard and soft blocks can provide a good CO_2_ separation performance without loss of its permeability.

Improved polymeric membrane materials with superior separation performance can be obtained by synthesizing new polymers or modification or blending existing commercial polymers with organic or inorganic compounds [208, 224].

Due to high operating cost of membrane processes, it is necessary to perform more researches and studies about preparation of suitable membranes.

#### 2.4.3. Mixed Matrix Membranes

Zeolites, carbon molecular sieves (CMS), and many polymeric materials offer attractive transport properties for CO_2_ separation. By mixing membrane material, excellent membrane with high performance for CO_2_ separation (selectivities of CO_2_/N_2_ = 17.8–39.6) can be prepared [200]. A group of scientists proposed the use of membrane based on polymer/immobilized liquid system especially polymerized ionic liquid membrane (PILM) or gelled ionic liquid membrane. ILMs consisting of aqueous solutions of 20% DEA immobilized in 25.4 *μ*m microporous polypropylene supports have low permeability and suitable selectivity (974 barrer, 276, resp.) in 2 atm at 298 K [225–228].

#### 2.4.4. Hollow Fiber Membrane

Most industrially important membranes for gas separations are hollow fiber ones. Asymmetric hollow fiber membranes (such as polyvinylidene difluoride (PVDF)) with inner skinless structures are favourable for CO_2_ separation and absorption in gas-liquid membrane by low mass-transfer resistance and high permeability. In addition, this process can achieve significantly high adsorption efficiencies due to the much larger surface area for gas-liquid interface than conventional gas absorption processes [206, 229–232].

According to data in Table 6, inorganic membranes have high permeability (about 150000 barrer) and low selectivity (about 15). Of course, some of inorganic membranes such as Y (FAU) with *α*-A1_2_O_3_ support and chitosan group are highly selective for CO_2_/N_2_ separation (selectivity (*α*) *≈* 100–800). Among polymeric membranes, polyamines have high permeability and selectivity (10^6^ (barrer) and 980, resp.), and the second FSCM membranes have high permeability and fine selectivity (10^5^ (barrer), 230, resp.). Other polymeric membrane groups are not selective for CO_2_/N_2_ separation, and maximum selectivity of these membranes is about 30.

### 2.5. Novel CO_**2**_ Capture Technologies

These methods include electrochemical pumps and chemical looping approaches to CO_2_ separation. The molten carbonate and aqueous alkaline fuel cells have been studied for use in separating CO_2_ from both air and flue gases. Electrochemical pumps discussed include carbonate and proton conductors. Molten carbonate is nearly 100% selective for CO_2_ separation, but major disadvantage in the application of molten carbonate electrochemical cells for CO_2_ separation is that this process is not repeatedly. Other disadvantages of these technologies are: corrosion, difficult operating condition (*T* = 873 K), and sensitivity to the presence of  SO_*x*_ [45, 233].

In chemical looping combustion, the oxygen for combustion of the fuel is provided by a regenerable metal oxide catalyst. The chemical looping scheme can be presented in the general form [45]:

(2) HC+metal  oxide ⟶CO2+H2O+lower  oxide  (and/or  metal)

(3) $$\begin{array}{c} \text{lower}\;\text{oxide}\left(\text{and}/\text{or}\;\text{metal}\right)+{\text{O}}_{2}⟶\text{metal}\;\text{oxide} \end{array}$$

Nickel oxide is one main candidate for the chemical looping combustion of methane, as low as 673 K, because it is extensive and effective for the chemical looping combustion [45].

### 2.6. Discussion

Various technologies such as absorption, adsorption, cryogenic distillation, and membrane have been suggested for CO_2_ separation from flue gases (Table 7). In this paper, various technologies for different feed conditions were investigated. Absorption is an important technology for CO_2_ separation. Although physical solvents required low energy for regeneration, they have low absorption capacity and selectivity for CO_2_ separation. Selexol is the best physical solvent and suitable for sweetening natural gas. However, physical absorption is not economical for flue gas streams with CO_2_ concentration lower than 15 vol% (95 US$/ton CO_2_ [234]).

Chemical solvents are classified in main groups such as alkanolamines, ammonia, aqueous piperazine (PZ), and amino acids. Chemical absorbents such as monoethanolamine (MEA) have high absorption capacities and are very flexible for CO_2_ separation; therefore, these solvents are usually preferred to physical solvents. Chemical absorption with alkanolamines is the only technology that is used in an industrial scale for post-combustion capture. Amines react rapidly, selectively, and reversibly with CO_2_ and are relatively nonvolatile and inexpensive solvents. In this process, the corrosion is the main problem; therefore, in recent studies, new amines and various mixtures of them were proposed and compared with previous ones to find suitable solvents. Suitable solvents for CO_2_ separation must have high CO_2_ absorption capacity, less corrosion, less viscosity and less regeneration energy. These studies showed that CASTOR 1 and 2, which are blended amine solvents (MEA/MDEA), are the best chemical adsorbents so far proposed for post-combustion CO_2_ capture. Experimental results indicated that amine amino acid salts (AAAS) have better performance than MEA of the same concentration for CO_2_ absorption, but do not deteriorate in the presence of oxygen. However, absorption has several disadvantages such as it requires high energy to regenerate solvents (3.0 GJ/ton CO_2_ for absorption with 40%wt MEA in 210 kPa [235]), therefore need more efforts in the future to reduce energy consumption in post-combustion CO_2_ capture with chemical absorption.

Adsorption is the one effective technology that can reduce energy and cost of the capture or separation of CO_2_ in post-combustion capture. Adsorption is suitable for separating CO_2_ from dilute and low flow rate stream, but flue gases conditions are the main problem against industrialization adsorption process. The CaO-MgAl_2_O_4_ and nano CaO/Al_2_O_3_ are the best chemical adsorbents. Although, the chemical adsorbents have high capacity and selectivity, but their regeneration is difficult. Physical adsorption is the most suitable for CO_2_ capture at high pressures and low temperatures. At higher pressure (above 4 bar) activated carbons are more efficient than zeolites. The energy and cost of adsorption for activated carbons are nearly half of that of zeolites. On the other hand, zeolites (particularly 13X and 5A) have high selectivity for CO_2_ separation. Generally, zeolite 5A may have better adsorption efficiency at co-adsorption of SO_2_, NO and CO_2_ than zeolite 13X.

In order to achieve more selective CO_2_ separation from flue gases, the modified adsorbent surface was considered. New adsorbents such as honeyncomb monolith, MOFs, CHAs (NaCHA and CaCHA), PMO (MCM and SBA) and MSPs (Na_2_SiO_4_) are suitable adsorbents for selective CO_2_ separation but they require more researches and studies. However, the development of suitable adsorbents with high CO_2_ adsorption capacity, which can be replaced absorption with chemical adsorbent, is still demanded.

Cryogenic distillation separation can be used for CO_2_ separation but its major disadvantage is the large amount of energy required to provide the refrigeration. Many new processes have been proposed for using cryogenic, but generally this technology is not suitable for post-combustion capture and is appropriate for oxy-fuel combustion method and CO_2_ separation from exhaust of cement industry (stream with high CO_2_ concentration).

The membrane separation method is a continuous, steady-state, clean and simple process for CO_2_ recovery. Since the pressure drop is driving force for membrane process, the flue gas stream must compress. Since compressing flue gas is very difficult and expensive, membrane separation is suitable for high pressure stream with high concentration (>10 vol%). Inorganic membrane have high thermal and chemical stability but their selectivity is lower than polymeric membranes. Although Y (FAU) with *α*-Al_2_O_3_ support and arginine salt chitosan are the best inorganic membrane, zeolite mambranes are suitable ones for CO_2_ separation. Polymeric membranes are very selective for CO_2_ separation but they have low thermal stability. Therefore, polymeric membranes are suitable for application in pre-combustion processes. Glassy polymeric membranes have higher selectivity, while the rubbery polymeric membranes have higher thermal stability. Perfect membrane with high performance for CO_2_ separation (selectivities of CO_2_/N_2_ = 17.8–39.6) can be prepared by mixing various membranes.

Because of operating problems and high cost of compressing, membrane separation is not suitable for post-combustion capture, but membrane technology is suitable for producing oxygen-enriched streams from air, in oxy-fuel combustion systems.

Electrochemical pumps and chemical looping are two new technologies suggested for CO_2_ capture. Now these technologies are not effective in comparison with other technologies. Therefore, application of electrochemical pumps and chemical looping in CCS needs more research.

## 3. Conclusion

Because of economical and environmental incentives, researchers have mainly focused on CO_2_ separation from different process streams, especially from the flue gases. In recent years, post-combustion capture has been the topic of many researches, because it is more flexible and can be easily added to the fossil fuel power plants.

Based on above findings, it can be concluded that flue gases properties (mainly concentration of CO_2_, temperature and pressure) are the most effective factors for selection of suitable process for CO_2_ separation.

Since flue gases have high temperature (about 373 K), low pressure, and low CO_2_ concentration (1 atm and 10–15% moL), bulk absorption and adsorption processes may be the best suitable process for CO_2_ separation from these streams. Due to simplicity of absorption process, this process has been applied in industrial plants, although many researches have been focused on preparation of adsorbents with high selectivity and capacity, in recent years. For industrial application, more studies about adsorbents are necessary. Cryogenic distillation and membrane processes are efficient for gas streams with high CO_2_ concentration. Therefore, these process are economically efficient for pre-combustion capture. In recent years, different studies have been performed to optimize cryogenic cycles and preparation of suitable membrane for CO_2_ separation from post-combustion flue gases.

By the result of this study, future research direction on the scale-up and industrialization of adsorption (with modified adsorbent), and membrane process for CO_2_ separation is suggested. Therefore more studies must be focused on modeling and simulation of these processes (membrane and adsorption), although research for finding new adsorbent, suitable mambrane (with mixed or modified present membrane) and blending amine solvents can reduce CCS cost.

## Figures and Tables

**Table 1:** The main greenhouse gases and their concentration [2, 3].

Compound	Preindustrial concentration (ppmv)	Concentration in 2011 (ppmv)	Atmospheric lifetime (years)	Main human activity source	GWP**
Carbon dioxide (CO_2_)	280	388.5	~100	Fossil fuels, cement production, land use	1
Methane (CH_4_)	0.715	1.87/1.748	12	Fossil fuels, rice paddies, waste dumps, livestock	25
Nitrous oxide (N_2_O)	0.27	0.323	114	Fertilizers, combustion industrial processes	298
CFC-12 (CCL_2_F_2_)	0	0.000533	100	Liquid coolants, foams	10,900
CF-113 (CCl_2_CClF_2_)	0	0.00000075	85	n.a.	6,130
HFC 23 (CHF_3_)	0	0.000018	270	Electronics, refrigerants	11,700
HCFC-22 (CCl_2_F_2_)	0	0.000218	12	Refrigerants	1,810
HFC 134 (CF_3_CH_2_F)	0	0.000035	14	Refrigerants	1,300
HCFC-141b (CH_3_CCl_2_F)	0	0.00000022	9.3	n.a.	725
HCFC-142b (CH_3_CClF_2_)	0	0.00000020	17.9	n.a.	2,310
HFC 152 (CH_3_CHF_2_)	0	0.0000039	1.4	Industrial processes	140
Perfluoromethane (CF_4_)	0.00004	0.00008*	50,000	Aluminum production	6,500
Perfluoroethane (C_2_F_6_)	0	0.000003*	10,000	Aluminum production	9,200
Sulfur hexafluoride (SF_6_)	0	0.00000712*	3,200	Dielectric fluid	22,800

*Concentration in 2011.

**Global warming potentials (GWPs) measure the relative effectiveness of GHGs in trapping the Earth's heat.

**Table 2:** Various solvents suggested for CO_2_ separation.

Group of solvents	Advantage	Disadvantage	Application	Reference
Physical				
Dimethyl ether of polyethylene glycol (Selexol)	(i) Require low energy for regeneration (less than 20% of the value for chemical absorbent) (ii) Low vapor pressure, low toxicity, and less corrosive solvent	(i) Dependent on temperature and pressure; therefore they are not suitable for post-combustion process (ii) Low capacity for CO_2_ absorption	Natural gas sweetening	[29, 39, 57, 62, 63]
Glycol			Capturing CO_2_ and H_2_S at higher concentration	
Glycol carbonate			Separating CO_2_ from other gases	
Methanol (Rectisol)			CO_2_ removal from various streams	
Fluorinated solvent			(i) CO_2_ removal from various streams (ii) Separating CO_2_ from other gases	

Chemical				
Alkanolamines: monoethanolamine (MEA), diethanolamine (DEA), and methyl diethanolamine (MDEA)	(i) React rapidly (ii) High selectively (between acid and other gases) (iii) Reversible absorption process (iv) Inexpensive solvent	(i) Low CO_2_ loading capacity (ii) Solvent degradation in existence of SO_2_ and O_2_ in flue gas (concentrations must be less than 10 ppm and 1 ppm) (iii) High equipment corrosion rate (iv) High energy consumption	Important for removing acidic components from gas streams	[58, 60, 61, 64–66]
Amino acid and aqueous amino acid salt	(i) The possibility of adding a salt functional group. (ii) The nonvolatility of solvents (iii) Having high surface tension (iv) Having better resistance to degradation than other chemical solvents (v) Better performance than MEA of the same concentration for CO_2_ absorption	Decreased performance in the presence of oxygen	Suggested for CO_2_ separation from flue gases	[65, 67–69]
Ammonia	(i) No degradation in the presence of SO_2_ and O_2_ in the flue gases (ii) No corrosion effect (iii) Require low energy to regeneration (1/3 that required with MEA) (iv) Low costs with aqueous ammonia, respectively, 15% and 20% less than with MEA	(i) Reversible at lower temperatures (not suitable for post-combustion) (ii) Production of solid products and their operating problems (iii) Explosion of dry CO_2_-NH_3_ reaction in the high concentration of CO_2_ in the flue gas (explosive limit for NH_3_ gas is 15–28%)	Suggested for CO_2_ separation from flue gases	[39, 70]
Ionic liquid (IL)	(i) Very low vapor pressure (ii) Good thermal stability (iii) High polarity (iv) Nontoxicity	Increased viscosity with CO_2_ absorption	Suggested for CO_2_ separation from flue gases	[71–74]
Aqueous piperazine (PZ)	(i) Fast absorption kinetics (CO_2_ absorption rate with aqueous PZ is more than double that of MEA) (ii) Low degradation rates for CO_2_ separation (iii) Negligible thermal degradation in concentrated PZ solutions (iv) Favorable equilibrium characteristics (v) Very low heat of absorption (10–15 kCal/mol CO_2_), 80–90% energy required for aqueous amine system	Lower oxidative degradation of concentrated PZ (i.e., 4 times slower than MEA in the presence of the combination of Fe^2+^/Cr^3+^/Ni^2+^ and Fe^2+^/V^5+^)	(i) Effective for treating syngas at high temperatures (ii) Application of additional amine promoters for natural gas treating and CO_2_ separation from flue gases	[29, 66, 75, 76]

**Table 3:** Adsorption capacity of chemical adsorbents for post-combustion CO_2_.

Sorbent	Operating temperature (K)	Operating pressure (kPa)	CO_2_ capture capacity (mol CO_2_/kg sorbent)	Regeneration cycles, *n*	CO_2_ capture capacity remained after *n* cycles (%)	Reference
Mesoporous (MgO)	298	101	1.8	3	100	[93]
CaO nanopods	873	101	17.5	50	61.1	[94]
CaO derived from nanosized CaCO_3_	923	101	16.7	100	22.2	[93]
CaO-MgAl_2_O_4_ (spinel nanoparticles)	923	101	9.1	65	84.6	[93]
Nano CaO/Al_2_O_3_	923	101	6.0	15	61.7	[93]
Lithium silicate nanoparticles	883	101	5.77	n.a.	n.a.	[93]
Nanocrystalline Li_2_ZrO_3_ particles	843	101	6.1	8	100	[93]
CaO/Al_2_O_3_	923	101	6.02	n.a.	n.a.	[93]
Lithium silicate	993	n.a.	8.18	n.a.	n.a.	[17]
Lithium zirconate	673	100	5.0	n.a.	n.a.	[93]
Lithium orthosilicate	873	100	6.13	n.a.	n.a.	[93]
Calcium oxide	873	100	17.3	n.a.	n.a.	[93]
Magnesium hydroxide	473	1034	3.0	n.a.	n.a.	[93]
Mesoporous magnesium oxide	373	100	2.27	n.a.	n.a.	[93]
Lithium Silicate nano particles	873	101	5	n.a.	n.a.	[95]
HTI-HNa	573	134	1.109	50	93.3	[93]

**Table 4:** Adsorption capacity of physical adsorbents for post-combustion CO_2_.

Sorbent	Operating temperature (K)	Operating pressure (kPa)	CO_2_ capture capacity (mol CO_2_/kg sorbent)	Regeneration cycles, *n*	CO_2_ capture capacity remained after *n* cycles (%)	Reference
Activated carbon	303	110	1.58	n.a.	n.a.	[93]
AC (4% KOH)	303	30	0.55	n.a.	n.a.	[93]
AC (EDA + EtOH)	303	30	0.53	n.a.	n.a.	[93]
AC (4% KOH + EDA + EtOH)	303	30	0.64	n.a.	n.a.	[45, 70, 79]
NiO-ACs	298	101	2.227	n.a.	n.a.	[104]
13X	393	15.198	0.7	n.a.	n.a.	[105]
5A	393	15.198	0.38	n.a.	n.a.	[105, 106]
4A	393	15.198	0.5	n.a.	n.a.	[105]
WEG-592	393	15.198	0.6	n.a.	n.a.	[105]
APG-II	393	15.198	0.38	n.a.	n.a.	[105]
Na-Y	273	10.132	4.9	n.a.	n.a.	[105]
Na-X	373	101.32	1.24	2	n.a.	[105]
NaKA	373	101.32	3.88	—	n.a.	[105]
NaX-h	323	101.32	2.52	2	n.a.	[105]
NaX-h	373	101.32	1.37	2	n.a.	[105]
Na-X-c	323	101.32	2.14	2	n.a.	[105]
Na-X-c	373	101.32	1.41	2	n.a.	[105]
Cs-X-h	323	101.32	2.42	2	n.a.	[105]
Cs-X-h	373	101.32	1.48	2	n.a.	[105]
Cs-X-c	323	101.32	1.76	2	n.a.	[105]
Cs-X-c	373	101.32	1.15	n.a.	n.a.	[105]
MCM-41	298	100	0.62	n.a.	n.a.	[93]
MCM-41 (DEA)	348	100	1.26	n.a.	n.a.	[93]
MCM-41 (50% PEI)	348	100	2.52	n.a.	n.a.	[93]
Activated carbon	303	30	0.35	n.a.	n.a.	[93]
MCM-41 (50% PEI) “molecular basket”	348	100	2.95	n.a.	n.a.	[93]
PE-MCM-41	298	100	0.50	n.a.	n.a.	[93]
PE-MCM-41 (TRI)	298	100	2.85	n.a.	n.a.	[93]
PE-MCM-41 (DEA)	348	100	2.36	n.a.	n.a.	[93]
MCM-48	298	100	0.033	n.a.	n.a.	[93]
MCM-48 (APTS)	298	100	0.639	n.a.	n.a.	[93]
MCM-41	298	100	0.62	n.a.	n.a.	[93]
Molecular basket' MCM-41 (50% PEI)	348	100	2.5	8	96.0	[93]
PE-MCM-41 (TRI)	298	100	1.8	10	94.4	[93]
PE-MCM-41 (DEA)	298	100	2.9	7	96.6	[93]
MWNT	303	101	1.7	20	n.a.	[4, 93]
Unmodified [(Cu_3_(btc)_2_]*	298	1818	6.7	n.a.	n.a.	[101]
CNT@ (Cu_3_(btc)_2_)	298	1818	13.52	n.a.	n.a.	[101]
MIL-101**	298	1010	0.84	n.a.	n.a.	[101]
MWCNT@MIL-101	298	1010	1.35	n.a.	n.a.	[101]
MOF-2	298	4545	3.20	n.a.	n.a.	[107]
MOF-177	298	4545	33.5	n.a.	n.a.	[107]
Zr-MOFs	273	988	8.1	n.a.	n.a.	[107]
Ca-Al LDH with ClO_4_^−^	406	1	3.55	n.a.	n.a.	[108]
Pd-GNP nanocomposite	298	1111	5.1	n.a.	n.a.	[109]
f-GNP	298	1111	4.3	n.a.	n.a.	[109]
Pd-GNP nanocomposite	298	1111	4.5	n.a.	n.a.	[109]
f-GNP	298	1111	3.8	n.a.	n.a.	[109]
Pd-GNP nanocomposite	298	1111	4.1	n.a.	n.a.	[109]
f-GNP	298	1111	3.3	n.a.	n.a.	[109]
Ceria-based oxides doped with 5% gallium (III)	298	101	0.282	n.a.	n.a.	[110]
Amine modified layered double hydroxides (LDHs)	298–353	101	0.74–1.75	n.a.	n.a.	[108]

*Cu_3_(btc)_2_; btc: 1,3,5-benzene-tricarboxylate.

**MIL-101 or Cr_3_(F,OH)(H_2_O)_2_O[(O_2_C)C_6_H_4_(CO_2_)]_3_ *·n*H_2_O (*n* ≈ 25) is one of the metal organic frameworks with Lewis acid sites that can be activated by removal of guest water molecules.

**Table 5:** Comparison between several adsorption cycles for CO_2 _separation process [166].

Process	CO_2_ feed molar fraction (%) (other gases present)	CO_2_ purity (%)	CO_2_ recovery (%)
PSA	13 (O_2_)	99.5	69
TSA	10	95	81
TSA	17	n.a.	40
ESA	10	23.33	92.57
VSA	15	90	90
VSA	17	n.a.	87
3-bed VSA	12	90–95	60–70
PSA/VSA	20	58–63	70–75
PSA/VSA	15 (H_2_O)	59	87
VPSA	17	99.5–99.8	34–69
VPSA	16 (O_2_)	99	53–70
PTSA	10	99	90
2-bed-2-step PSA	n.a.	18	90
VTSA	17	n.a.	97

**Table 6:** Carbon dioxide and nitrogen gas permeability data for different membranes.

Name	Feed pressure (atm)	Temperature (K)	P* (CO_2_) (barrer)	P* (N_2_) (barrer)	*α* (CO_2_/N_2_)	Reference
Ion-exchanged zeolites membrane						
Y (FAU) with *α*-A1_2_O_3_ support	n.a.	308	n.a.	n.a.	139	[197]
ZSM-5 (MFI) with *α*-A1_2_O_3 _support	n.a.	n.a	n.a.	n.a.	3	[197]
ZSM-5/polymeric silica	n.a.	373	1140	n.a.		[198]
Stainless steel support infiltrated with a eutectic molten carbonate mixture (Li/Na/K)	n.a.	923	7780	n.a.	16	[199]
Y-type	n.a.	303–403	35900–89800	n.a.	5	[200]
NaY	n.a.	313	359000	n.a.	5	[200]
Li(20%)Y	n.a.	308	210000	n.a.	3	[200]
K(30%)Y	n.a.	308	269000	n.a.	9	[200]
K(62%)Y	n.a.	313	150000	n.a.	6	[200]
Rb(38%)Y	n.a.	313	150000	n.a.	3	[200]
Cs(32%)Y	n.a.	313	59900	n.a.	2	[200]
20% K_2_CO_3_, 80% Li_2_CO_3_	n.a.	798	2990	n.a.	4	[199]
MCM-48	n.a.	n.a.	10200	n.a.	0.8	[189]
PEI-modified MCM-48	n.a.	363	14100	n.a.	80	[201]
Chitosan	1.75	295	100	n.a.	100	[192]
Swollen chitosan	1.5	383	482	n.a.	250	[192]
Arginine salt-chitosan	1.5	383	1500	n.a.	852	[194]

Polyacetylene						
Polytrimethyl-prop-1-ynyl-silane	n.a.	298	19000	1800	10.6	[193]
Poly-3,3-dimethyl-but-1-yne	n.a.	298	560	43	13.0	[193]
Poly-1-(dimethyl-trimethylsilanylmethyl-silanyl)-propyne	n.a.	298	310	21	14.8	[193]
Poly-1-[dimethyl-(2-trimethylsilanyl-ethyl)-silanyl]-propyne	n.a.	298	150	14	10.7	[193]
Polytrimethyl-(2-prop-1-ynyl-phenyl)-silane	n.a.	298	290	24	12.1	[193]
Poly-1-prop-1-ynyl-2-trifluoromethyl-benzene	n.a.	298	130	7.3	17.8	[193]
Poly-dec-2-yne	n.a.	298	130	14	9.3	[193]
Poly-1-chloro-dec-1-yne	n.a.	298	170	16	10.6	[193]
Poly-1-chloro-oct-1-yne	n.a.	298	130	11	11.8	[193]
Poly-1-chloro-hex-1-yne	n.a.	298	180	10	18	[193]
Polyhexyl-dimethyl-prop-1-ynyl-silane	n.a.	298	71	4.3	16.5	[193]
Polytrimethyl-(1-pentyl-prop-2-ynyl)-silane	n.a.	298	120	8.7	13.8	[193]
Polyhexyl-dimethyl-(1-propyl-prop-2-ynyl)-silane	n.a.	298	70	6.3	11.1	[193]
Polyprop-1-ynyl-benzene	n.a.	298	25	2.2	11.4	[193]
Polybut-1-ynyl-benzene	n.a.	298	40	4.5	8.9	[193]
Polyoct-1-ynyl-benzene	n.a.	298	48	5.5	8.7	[193]
Polychloroethynyl-benzene	n.a.	298	23	1.0	23.0	[193]
Poly-1-ethynyl-2-methyl-benzene	n.a.	298	15	3.0	5.0	[193]
Polydimethyl-phenyl-(1-propyl-prop-2-ynyl)-silane	n.a.	298	54	2.5	21.6	[193]

Polyarylene ether						
6FPT-6FBPA	1.0	308	25.29	2.18	11.6	[193]
6FPT-BPA 1.0 35	1.0	308	18.53	1.37	13.5	[193]
6FPPy-6FBPA	1.0	308	29.46	2.39	12.32	[193]
6FPPy-BPA	1.0	308	21.44	1.70	12.6	[193]

Fixed site carrier membrane (FSCM)						
Polarix	2.0	303	10^7^	n.a.	50	[202]
PAAM-PVA/PS	10	298	2.4 × 10^5^	n.a.	80	[203]
PVAm/PVA blend	1.45	298	2.12 × 10^6^	n.a.	145	[204]
PEI/PVA	n.a.	298	10^4^	n.a.	230	[184]
PDMA/PS	2	296	3 × 10^5^	n.a.	53	[143]

Polyamine						
PA12	10	308	120	n.a.	51	[152]
PA6	10	308	66	n.a.	56	[152]
Polyethyleneimine/polyvinyl butyral	0.132	318	380	n.a.	32	[193]
Poly[(2-N,N-dimethyl) aminoethyl methacrylate]	0.237	298	370	n.a.	111	[193]
Poly(vinylbenzyltrimethyl ammonium fluoride)	0.224	296	113	n.a.	983	[193]
Polyethyleneimine/poly(vinyl alcohol)	0.355	298	650	n.a.	235	[193]
PEI/PDMS/PEBA1657/PDMS	5	298	1.57 × 10^6^	n.a.	64	[205]

Polyarylate						
BPA/IA	10	308	5.4	0.24	22.5	[193]
BPA/tBIA	10	308	24.2	1.20	20.2	[193]
HFBPA/IA	10	308	19.1	1.11	17.2	[193]
HFBPA/tBIA	10	308	56.9	3.88	14.7	[193]
PhTh/IA	10	308	6.74	0.28	24.1	[193]
PhTh/tBIA	10	308	23.8	1.09	21.8	[193]
FBP/IA	10	308	12.4	0.57	12.4	[193]
FBP/tBIA	10	308	36.8	1.93	19.1	[193]
TBBPA/IA	10	308	4.93	0.18	27.4	[193]
TBBPA/tBIA	10	308	21.5	0.90	23.9	[193]
TBHFBPA/IA	10	308	25.6	1.07	23.9	[193]
TBHFBPA/tBIA	10	308	85.1	4.47	19.0	[193]
TBPhTh/IA	10	308	8.34	0.29	28.8	[193]
TBPhTh/tBIA	10	308	30.6	1.28	23.9	[193]
TBFBP/IA	10	308	20.4	0.70	29.1	[193]
TBFBP/tBIA	10	308	69.5	2.94	23.6	[193]
DMBPA/IA	10	308	1.24	0.063	19.7	[193]
DMBPA/Tbia	10	308	8.0	0.39	20.5	[193]
TMBPA/IA	10	308	12.0	0.58	20.7	[193]
TMBPA/tBIA	10	308	44.6	2.52	17.7	[193]
DiisoBPA/IA	10	308	5.16	0.27	19.1	[193]
DiisoBPA/tBIA	10	308	16.1	1.08	14.9	[193]
DBDMBPA/IA	10	308	5.45	0.22	24.8	[193]
PhAnth/IA	10	308	9.0	0.36	25	[193]
PhAnth/tBIA	10	308	25.9	1.35	19.2	[193]
FBP/IA	10	308	12.4	0.57	21.8	[193]
FBP/tBIA	10	308	36.8	1.93	19.1	[193]

Polycarbonates						
PC	1–10	308	6.0–6.8	0.289–0.32	21	[193]
TMPC	1–10	308	17.58–18.6	1.0	18.6	[193]
TCPC	1	308	6.66	0.36	18.5	[193]
TBPC	1	308	4.23	0.182	23.2	[193]
HFPC	10	308	24	1.6	15.0	[193]
TMHFPC	10	308	111	7.4	15.0	[193]
NBPC	10	308	9.1	0.47	19.4	[193]
PCZ	10	308	2.2	0.105	21.0	[193]
PC-AP	2	308	9.48	0.361	26.3	[193]
FBPC	2	308	15.1	0.592	25.5	[193]

Polyethylene oxide						
PEO	7.8	298	8.1	0.07	140	[193]
PEO	4.4–14.6	308–318	13–52	0.24–1	55	[193]
PEO-PBT	n.a.	308	120	2	60	[193]
EO/EM/AGE (80/20/2)	n.a.	308	773	16.8	46	[193]
EO/EM/AGE (77/23/2.3)	n.a.	308	680	15.5	44	[193]
EO/EM/AGE (96/4/2.5)	n.a.	308	580	12.1	48	[193]

Polyimides						
Amine modified polyimide	0.368	308	186	n.a.	38	[193]
PMDA-BAPHF	6.8	308	11.8	0.66	17.8	[193]
PMDA-3BAPHF	6.8	308	6.12	0.29	21.1	[193]
PMDA-4,4′-ODA	6.8–10	308	1.14–2.7	0.049–0.1	23.3	[193]
PMDA-3,3′-ODA	6.8–10	308	0.50–3.55	0.018–0.145	24.5–27.8	[193]
PMDA-MDA	10	308	4.03	0.20	20.2	[193]
PMDA-IPDA	10	308	29.7	1.50	19.8	[193]
PMDA-BAPHF	10	308	17.6	0.943	18.7	[193]
PMDA-BATPHF	10	308	24.6	1.50	16.4	[193]
BPDA-BAHF	1–10	298–308	23–27.7	0.6–1.39	19.9–37.7	[193]
BPDA-mTrMPD	10	308	137	8.42	16.3	[193]
BTDA-4,4-ODA	10	308	0.625	0.0236	26.5	[193]
BTDA-BAPHF	10	308	4.37	0.195	22.4	[193]
BTDA-BAHF	10	308	10.1	0.45	22.4	[193]
BTDA-mTrMPD	10	308	30.9	1.55	19.9	[193]
BTDA-BAFL	1	298	15	0.39	38.5	[193]
PI	10	308	2.00	0.063	31.7	[193]
oMeCat-durene	1	303	27	0.83	33	[193]
mMeCat-durene	1	303	20	0.59	34	[193]
DMeCat-durene	1	303	63	2.05	31	[193]
mtBuCat-durene	1	303	71	2.55	28	[193]
oMeptBuCat-durene	1	303	67	2.5	27	[193]
TMeCat-durene	1	303	200	8.1	25	[193]
mMetCat-MDA	1	303	22	0.65	34	[193]
mtBuCat-MDA	1	303	63	2.2	29	[193]
TMeCat-MDA	1	303	110	3.8	30	[193]
TMeCat-TMB	1	303	39	1.2	33	[193]
DBuCat-TMB	1	303	95	4.9	19	[193]
mtBuCat-DMOB	1	303	6.7	0.21	32	[193]
TMeCat-6FiPDA	1	303	54	1.9	28	[193]
6F	3	n.a.	114	5.8	19.6	[193]
TMMPD	3	n.a.	600	35.1	17.1	[193]
IMDDM	3	n.a.	196	10.8	18.1	[193]
ODA	3	n.a.	25	0.97	25.8	[193]
Matrimid 5218	10	308	6.5	0.25	25.6	[193]

6FDA-based polyimides						
6FDA-pPDA	10	308	15.3	0.80	19.12	[193]
6FDA-pDiMPDA	10	303	42.7	2.67	16.0	[193]
6FDA-durene	10	308	440	35.60	12.4	[193]
6FDA-durene	10	303	456	35.50	12.85	[193]
6FDA-mPDA	6.8–10	308	8.23–9.20	0.36–0.447	20.6–22.7	[193]
6FDA-mMPDA	6.8–10	303	40.1–42.5	2.12–2.24	17.9–20.1	[193]
6FDA-mTrMPDA	10	308	431	31.6	13.6	[193]
6FDA-DATr	6.8	303	28.63	1.31	21.9	[193]
6FDA-DBTF	6.8	308	21.64	1.17	18.5	[193]
6FDA-PHDoeP	6.8	303	8.59	4.50	1.91	[193]
6FDA-PEPE	6.8	308	6.88	0.255	27.0	[193]
6FDA-PBEPE	6.8	303	2.50	0.099	25.3	[193]
6FDA-PMeaP	6.8	308	2.41	0.086	28.0	[193]
6FDA-3,4′ODA	10	303	6.11	0.259	23.6	[193]
6FDA-APAP	10	308	10.7	0.473	22.6	[193]
6FDA-pp′ODA	10	303	16.7	0.733	22.8	[193]
6FDA-BAPHF	10	308	19.1	0.981	19.5	[193]
6FDA-BATPHF	10	303	22.8	1.30	17.5	[193]
6FDA-BAHF	10	308	51.2	3.11	16.5	[193]
6FDA-1,5-NDA	10	308	23	1.1	21	[193]
6FDA-durene 24 h amidation	10	n.a.	11.6	1.33	8.75	[193]
6FDA-durene/mPDA (50/50)	10	n.a.	84.6	5.18	16.4	[193]
6FDA-durene/mPDA (50/50) 4 h amidation	10	n.a.	54.9	3.38	16.2	[193]
6FDA-durene/mPDA (50/50) 6 h amidation	10	n.a.	49.1	3.27	15.0	[193]
6FDA-durene/mPDA (50/50) 12 h amidation	10	n.a.	46.0	2.94	15.6	[193]
6FDA-durene/mPDA (50/50) 24 h amidation	10	n.a.	36.0	2.06	17.5	[193]
6FDA-durene/mPDA (50/50) 48 h amidation	10	n.a.	24.5	1.38	17.8	[193]
6FDA-FDA/HFBAPP (1/1)	1.1 kg/cm^2^	303	465.0	19.9	23.4	[193]
6FDA-ODA	10	308	23	0.83	27.7	[193]
6FDA-4,4-ODA	6.8	303	22.0	0.94	23.4	[193]
6FDA-MDA	10	308	19	0.81	23.5	[193]
6FDA-4BDAF	6.8	303	19	0.98	19.4	[193]
6FDA-3,3′-ODA	6.8	308	2.1	0.10	21	[193]
6FDA-3BDAF	6.8	303	6.3	0.24	26.3	[193]
6FDA-IPDA	10	308–328	24.3–27.4	0.87–1.39	19.7–27.9	[193]
6FDA-DAF	10	308–328	19.5–21.3	0.81–1.15	18.5–24.1	[193]
PI-1	1	303	32	1.4	22.9	[193]
PI-3	1	303	360	16.5	21.8	[193]
PI-4	1	303	62	2.4	25.8	[193]
PI-5	1	303	190	7.3	26.0	[193]
6FDA-BAFL	1	298	98	3.3	29.7	[193]

Poly(phenylene oxide)						
PPO (hollow fiber)	4	308	10^6^		21	[205]
PPS	1.5	308	1.60	0.046	34.8	[193]
PDMPO	1.5	308	65.5	3.5	18.7	[193]
PDPPO	1.5	308	39.9	1.5	26.6	[193]
PDMPO	6.891	295	90.0	3.7	24.3	[193]
PDMPO (20.0% brominated)	6.891	295	93.6	3.8	24.6	[193]
PDMPO (37.4% brominated)	6.891	295	97.1	3.7	26.2	[193]
PDMPO (60.0% brominated)	6.891	295	159.9	8.0	20.0	[193]

Polypyrrole						
6FDA-TAB	10	308	54.0	2.6	20.8	[193]
6FDA-TADPO	10	308	27.6	1.2	23.0	[193]
BBL	10	308	0.12	0.003	46.3	[193]

Polysulfones						
PSF	10	308	5.6	0.25	22.4	[193]
TMPSF	10	308	21	1.06	19.8	[193]
HFPSF	10	308	12	0.67	17.9	[193]
TMHFPSF	10	308	72	4.0	18	[193]
PSF-F	10	308	4.5	0.20	22.5	[193]
PSF-O	10	308	4.3	0.20	21.5	[193]
PSF-P	10	308	6.8	0.32	21.3	[193]
TMPSF-F	10	308	5.5	0.61	9.0	[193]
TMPSF-P	10	308	13.2	0.57	23.2	[193]
BIPSF	10	308	5.6	0.24	23.3	[193]
TMBIPSF	10	308	31.8	1.21	26.3	[193]
1,5-NPSF	10	308	1.6	0.057	28.1	[193]
2,6-NPSF	10	308	1.5	0.051	29.4	[193]
2,7-NPSF	10	308	1.8	0.074	24.3	[193]
DMPSF	10	308	2.1	0.091	23.1	[193]
HMBIPSF	10	308	25.5	1.2	23.3	[193]
DMPSF-Z	10	308	1.4	0.057	24.6	[193]
PSF-AP	2	308	8.12	0.278	29.2	[193]
FBPSF	2	308	13.8	0.484	28.5	[193]
PSF-M	1	308	2.8	0.11	25.5	[193]
TMPSF-M	10	308	7.0	0.28	25.0	[193]
PSF-BPFL	1	308	10	0.25	40	[193]
3,4′-PSF	1	308	1.5	0.066	22.7	[193]
1,3-ADM PSF	35	308	7.2	0.33	21.8	[193]
2,2-ADM PSF	35	308	9.5	0.46	20.6	[193]
PSF (6% Br, 92% C*≡*CSiMe_3_)	1	308	36.5	2.1	17.4	[193]
PSF (3% Br, 47% C*≡*CSiMe_3_)	1	308	18.5	1.24	14.9	[193]
PSF (21% Br, 77% C*≡*CSiMe_3_)	1	308	28.2	1.7	16.6	[193]
PSF (5% Br, 45% C*≡*CSiMe_3_)	1	308	16.4	0.9	18.2	[193]
PSF	1	308	5.6	0.25	22.4	[193]
PSF-*s*-HBTMS	1	308	21	0.96	22.2	[193]
PSF-*o*-HBTMS	1	308	70	3.29	21.3	[193]
PSF-CH2-TMS	1	308	18	0.95	18.9	[193]
EM3	1	308	29	1.3	22	[193]
EM2	1	308	6.2	0.24	26	[193]
EM1	1	308	4.8	0.16	30	[193]
SM3 (degree of substitution = 2.0)	1	308	18	0.77	23	[193]
SM3 (degree of substitution = 1.0)	1	308	10	0.38	26	[193]
SM1	1	308	5.1	0.17	30	[193]
PPSF	1	308	3.2	0.10	32	[193]
RM3	1	308	27	1.9	14	[193]
RM2	1	308	6.7	0.60	11	[193]
RM1	1	308	6.9	0.61	11	[193]
HFPSF	1	308	12.0	0.67	17.9	[193]
HFPSF-*o*-HBTMS	1	308	105	5.63	18.6	[193]
HFPSF-*s*-TMS	1	308	41	2.0	20	[193]
HFPSF-*o*-TMS	1	308	84	4.7	18	[193]
HFPSF-TMS	1	308	110	6.3	18	[193]
TM6FPSF	1	308	72	4.0	18	[193]
TM6FPSF-*s*-TMS	1	308	96	5.2	19	[193]
TMPSF-TMS	1	308	32	1.51	21.3	[193]
TMPSF-*s*-TMS	1	308	66.3	3.07	21.6	[193]
TMPSF-HBTMS	1	308	72	3.36	21.4	[193]

Other membranes						
HQDPA-PDA	7	303	0.598	0.016	37.4	[193]
HQDPA-PDA	7	373	1.70	0.111	15.3	[193]
HQDPA-DBA	7	303	0.683	0.015	45.5	[193]
HQDPA-DBA	7	373	2.10	0.125	16.8	[193]
HQDPA-MDBA	7	303	1.18	0.034	34.7	[193]
HQDPA-MDBA	7	373	2.37	0.160	14.8	[193]
HQDPA-EDBA	7	303	2.26	0.077	29.4	[193]
HQDPA-EDBA	7	373	4.18	0.292	14.3	[193]
12H	5	308	4.6	0.21	21.9	[193]
6H6F	5	308	8.6	0.44	19.5	[193]
6F6H	5	308	8.9	0.42	21.2	[193]
12F	5	308	12.9	0.76	17.0	[193]
PBK	10	308	3.3	0.13	25.4	[193]
PBK-S	10	308	3.27	0.11	29.7	[193]
PBSF	10	308	10.8	0.47	23.0	[193]
PES/PI	4	308	1.15 × 10^5^	n.a.	30	[193]
PPES	n.a.	273	0.92	0.027	34	[193]
PPESK	n.a.	273	0.75	0.042	18	[193]
20 percent DEA immobilized in 25.4 *μ*m microporous polypropylene supports	0.16–1.67	298	974–4825	n.a.	56–276	[200]

Copolymers and polymer blend						
PEBA 2533 (hollow fiber)	6.8	273	260	n.a.	32	[206]
PEBA/PSF composite	3.4	273	6.1 × 10^5^	n.a.	30	[206]
COPNA	n.a.	373	2990	n.a.	14	[200]
Pebax	n.a.	303	73	n.a.	15.6	[207]
Pebax/PEG10	n.a.	303	75	n.a.	15.8	[207]
Pebax/PEG20	n.a.	303	80	n.a.	15.9	[207]
Pebax/PEG30	n.a.	303	105	n.a.	15.1	[207]
Pebax/PEG40	n.a.	303	132	n.a.	15.1	[207]
Pebax/PEG50	n.a.	303	151	n.a.	15.5	[207]
Pebax/PEG-DME10	n.a.	303	123	n.a.	44	[208]
Pebax/PEG-DME20	n.a.	303	206	n.a.	45	[208]
Pebax/PEG-DME30	n.a.	303	300	n.a.	46	[208]
Pebax/PEG-DME40	n.a.	303	440	n.a.	42	[208]
Pebax/PEG-DME50	n.a.	303	606	n.a.	43	[208]
6FDA-TAB	10	308	54.0	2.8	19.3	[193]
6FDA/PMDA-TAB (50 : 50)	10	308	15.8	0.70	22.6	[193]
6FDA/PMDA-TAB (25 : 75)	10	308	3.13	0.098	31.9	[193]
6FDA/PMDA-TAB (10/90)	10	308	1.11	0.036	30.8	[193]
6FDA-TAB/DAM (75/25)	3	308	73.7	3.1	23.8	[193]
6FDA-TAB/DAM (50/50)	3	308	155	6.6	23.5	[193]
6FDA-DAM	3	308	370	29.5	12.5	[193]
6FDA/TMPDA	n.a.	308	400	23.5	17.02	[193]
6FDA/PMDA (1 : 6)-TMMDA (CH_2_Cl_2_ cast)	10	308	187	11.7	16.0	[193]
6FDA/PMDA (1 : 6)-TMMDA (NMP cast)	10	308	144	8.76	16.4	[193]
6FDA/PMDA (1 : 6)-TMMDA (DMF cast)	10	308	88.6	5.16	17.2	[193]
MDI-BPA/PEG (75)	2	308	31	0.70	44	[193]
MDI-BPA/PEG (80)	2	308	48	1.0	47	[193]
MDI-BPA/PEG (85)	2	308	59	1.20	49	[193]
L/TDI (20)-BPA/PEG (90)	2	308	47	0.92	51	[193]
L/TDI (40)-BPA/PEG (85)	2	308	35	0.72	48	[193]
IPA-ODA/PEO3 (80)	2	308	58	1.1	53	[193]
BPDA-pp′ODA	n.a.	303	18000	n.a.	31	[155]
BPDA-ODA/DAT (oxidized)	n.a.	308	599	n.a.	40	[155]
BPDA-ODA/DABA/PEO1 (75)	2	308	2.7	0.048	56	[193]
BPDA-mDDS/PEO1 (80)	2	308	3.8	0.066	58	[193]
BPDA-ODA/DABA/PEO2 (70)	2	308	14	0.25	57	[193]
BPDA-ODA/DABA/PEO2 (80)	2	308	36	0.64	56	[193]
BPDA-ODA/PEO3 (75)	2	308	75	1.4	52	[193]
BPDA-mDDS/PEO3 (75)	2	308	72	1.4	53	[193]
BPDA-mPD/PEO4 (80)	2	308	81	1.5	54	[193]
BPDA-ODA/PEO4 (80)	2	308	117	2.3	51	[193]
PMDA-ODA/DABA/PEO1 (80)	2	308	14	0.27	52	[193]
PMDA-ODA/PEO2 (75)	2	308	40	0.74	54	[193]
PMDA-mPD/PEO3 (80)	2	308	99	2.0	50	[193]
PMDA-APPS/PEO3 (80)	2	308	159	3.1	51	[193]
PMDA-APPS/PEO4 (70)	2	308	136	2.6	53	[193]
PMDA-mPD/PEO4 (80)	2	308	151	2.9	52	[193]
PMDA-ODA/PEO4 (80)	2	308	167	3.2	52	[152]
PMDA-pDDS/PEO4 (80)	2	308	238	4.9	49	[152]
PMDA/BTDA-BAFL (50 : 50)	1	298	43	1.3	33	[193]
PMDA/BTDA–BAFL (90 : 10)	1	298	130	3.8	34	[193]
BPDA-BAFL/HMDA (50 : 50)	1	298	0.54	0.014	39	[193]
PPES	n.a.	298	0.92	0.027	34	[193]
PPES/PPEK (3 : 1)	n.a.	298	2.94	0.074	40	[193]
PPES/PPEK (1 : 1)	n.a.	298	4.12	0.089	46	[193]
PPES/PPEK (1 : 3)	n.a.	298	2.06	0.026	39	[193]
PPES/PPEK (1 : 4)	n.a.	298	1.77	0.052	34	[193]
PPEK 18	n.a.	298	0.75	0.042	18	[193]
HQDPA-DPA/MDPA	7	303	0.957	0.023	41.2	[193]
HQDPA-DPA/MDPA	7	373	2.34	0.147	15.9	[193]
HQDPA-DPA/EDPA	7	303	1.334	0.036	37.6	[193]
HQDPA-DPA/EDPA	7	373	3.25	0.207	15.7	[193]
PI	10	308	2.00	0.063	31.7	[193]
PI/10PS	10	308	2.33	0.085	27.4	[193]
PI/15PS	10	308	2.32	0.09	25.8	[193]
PI/20PS	10	308	2.90	0.91	3.19	[193]
PI/25PS	10	308	4.29	0.91	4.71	[193]
PI/10PSVP	10	308	3.58	0.13	28.4	[193]
PI/15PSVP	10	308	3.71	0.14	26.5	[193]
PI/20PSVP	10	308	5.65	0.15	38.4	[193]
PI/25PSVP	10	308	6.55	1.55	4.31	[193]
NTDA-BDSA (30)/CARDO/ODA	3	303	70	1.7	41	[193]
NTDA-BDSA (30)/CARDO]	3	303	164	4.5	36	[193]
NTDA-BDSA (30)/BAPHF	3	303	23	0.64	36	[193]
NTDA-BDSA (30)/ODA	3	303	5.2	0.1	52	[193]
6FDA-FDA/HFBAPP (1/1)	1.1 kg/cm^2^	303	465	19.9	23.4	[193]
6FDA-durene/pPDA (80/20)	10	308	230	16.88	13.62	[193]
6FDA-durene/pPDA (50/50)	10	308	126	7.74	16.28	[193]
6FDA-durene/pPDA (20/80)	10	308	59.26	2.81	21.09	[193]
6FDA-durene/3,3′-DDS (75/25)	10	308	84.7	5.91	14.3	[193]
6FDA-durene/3,3′-DDS (50/50)	10	308	19.8	1.09	18.2	[193]
6FDA-durene/3,3′-DDS (25/75)	10	308	5.12	0.26	19.7	[193]
6FDA-3,3′-DDS	10	308	1.84	0.08	22.7	[193]
6FDA-6FpDA-DABA-12.5	4	308	34.0	2.01	16.9	[193]
6FDA-6FpDA–DABA-12.5 annealed	4	308	70.8	4.50	15.7	[193]
6FDA-6FpDA-DABA-12.5 (22.5% TMOS)	4	308	30.9	1.70	18.2	[193]
6FDA-6FpDA-DABA-12.5 (22.5% TMOS) annealed	4	308	47.6	3.16	15.1	[193]
6FDA-6FpDA-DABA-12.5 (15.0% MTMOS)	4	308	44.0	2.53	17.4	[193]
6FDA-6FpDA-DABA-12.5 (15.0% MTMOS) annealed	4	308	110	7.07	15.6	[193]
6FDA-6FpDA-DABA-12.5 (15.0% PTMOS) 4 35	4	308	32.3	1.80	17.9	[193]
6FDA-6FpDA-DABA-12.5 (15.0% PTMOS) annealed	4	308	91.8	5.59	16.4	[193]
6FDA-6FpDA-DABA-12.5 (22.5% PTMOS)	4	308	30.7	1.88	16.3	[193]
6FDA-6FpDA-DABA-12.5 (22.5% PTMOS) annealed	4	308	90.9	5.87	15.5	[193]
6FDA-6FpDA-DABA-25	4	308	20.3	1.20	16.9	[193]
6FDA-6FpDA-DABA-25 annealed	4	308	77.3	4.85	15.9	[193]
6FDA-6FpDA-DABA-25 (22.5% TMOS)	4	308	15.7	1.06	14.8	[193]
6FDA-6FpDA-DABA-25 (22.5% TMOS) annealed	4	308	79.8	4.87	16.4	[193]
6FDA-6FpDA-DABA-25 (15.0% MTMOS)	4	308	16.6	1.07	15.5	[193]
6FDA-6FpDA-DABA-25 (15.0% MTMOS) annealed	4	308	81.1	5.07	16.0	[193]
6FDA–6FpDA-DABA-25 (22.5% MTMOS)	4	308	16.6	1.07	15.5	[193]
6FDA-6FpDA-DABA-25 (22.5% MTMOS) annealed	4	308	60.1	3.837	15.7	[193]
6FDA-6FpDA-DABA-25 (15.0% PTMOS)	4	308	18.4	0.94	19.6	[193]
6FDA-6FpDA-DABA-25 (15.0% PTMOS) annealed	4	308	104	6.25	16.6	[193]
6FDA-6FpDA-DABA-25 (22.5% PTMOS)	4	308	19.1	0.98	19.5	[193]
6FDA-6FpDA-DABA-25 (22.5% PTMOS) annealed	4	308	104	6.25	16.6	[193]
Poly(5 : 5 BPA/BN)	5	308	5.71	0.19	30.1	[193]
Poly(7 : 3 BPA/BN)	5	308	4.62	0.16	28.9	[193]

Cross-linking polymers						
Poly(ethylene oxide-co-epichlorohydrin) (1 : 1) 1.1%	300	298	15.0	2.3	6.52	[193]
Poly(ethylene oxide-co-epichlorohydrin) (1 : 1) 2%	300	298	14.9	1.0	14.9	[193]
Poly(ethylene oxide-co-epichlorohydrin) (1 : 1) 5%	300	298	16.1	0.5	32.2	[193]
DM14/MM9 (100/0)	0.967	298	45	0.66	68	[193]
DM14/MM9 (100/0)	0.967	323	107	2.8	38	[193]
DM14/MM9 (90/10)	0.967	298	62	0.90	69	[193]
DM14/MM9 (90/10)	0.967	323	133	3.4	39	[193]
DM14/MM9 (70/30)	0.967	298	96	1.5	66	[193]
DM14/MM9 (70/30)	0.967	323	195	5.4	36	[193]
DM14/MM9 (50/50)	0.967	298	144	2.25	64	[193]
DM14/MM9 (50/50)	0.967	323	260	7.2	36	[193]
DM14/MM9 (30/70)	0.967	298	210	3.3	63	[193]
DM14/MM9 (30/70)	0.967	323	350	10.6	33	[193]
DB30/MM9 (100/0)	0.967	298	93	1.5	63	[193]
DB30/MM9 (100/0)	0.967	323	200	5.7	35	[193]
DB30/MM9 (90/10)	0.967	298	105	1.6	64	[193]
DB30/MM9 (90/10)	0.967	323	210	5.8	36	[193]
DB30/MM9 (70/30)	0.967	298	141	2.1	67	[193]
DB30/MM9 (70/30)	0.967	323	270	7.7	35	[193]
DB30/MM9 (50/50)	0.967	298	179	2.9	62	[193]
DB30/MM9 (50/50)	0.967	323	330	9.7	34	[193]
DB30/MM9 (30/70)	0.967	298	250	4.2	60	[193]
DB30/MM9 (30/70)	0.967	323	410	12.4	33	[193]
DM9/MM9 (90/10)	0.967	298	18.3	0.3	68	[193]
DM9/MM9 (90/10)	0.967	323	51	1.3	38	[193]
DM23/MM9 (90/10)	0.967	298	145	2.2	66	[193]
DM23/MM9 (90/10)	0.967	323	290	7.6	38	[193]
DB10/MM9 (90/10)	0.967	298	6.7	0.11	61	[193]
DB10/MM9 (90/10)	0.967	323	27	0.79	34	[193]
DB69/MM9 (90/10) (cooling)	0.967	298	240	4.3	56	[193]
DB69/MM9 (90/10) (cooling)	0.967	323	510	14.2	36	[193]
DB69/MM9 (90/10) (heating)	0.967	298	98	1.6	62	[193]
DB69/MM9 (90/10) (heating)	0.967	323	400	11.4	35	[193]
DM14/MM23 (30/70) (cooling)	0.967	298	240	3.9	62	[193]
DM14/MM23 (30/70) (cooling)	0.967	323	420	12	35	[193]
DM14/MM23 (30/70) (heating)	0.967	298	250	4.0	62	[193]
Matrimid 5218	10	308	6.5	0.25	25.6	[193]
Matrimid 5218, 1-day cross-linking	10	308	7.4	0.29	25.6	[193]
Matrimid 5218, 3-day cross-linking	10	308	6.0	0.24	25.2	[193]
Matrimid 5218, 7-day cross-linking	10	308	5.1	0.21	24.6	[193]
Matrimid 5218, 14-day cross-linking	10	308	4.7	0.19	24.1	[193]
Matrimid 5218, 21-day cross-linking	10	308	3.4	0.15	22.2	[193]
Matrimid 5218, 32-day cross-linking	10	308	1.9	0.13	15.0	[193]
6FDA-durene, 5 min cross-linked	10	308	136	11.1	12.3	[193]
6FDA-durene, 10 min cross-linked	10	308	91.8	6.53	14.1	[193]
6FDA-durene, 15 min cross-linked	10	308	70.0	6.05	11.6	[193]
6FDA-durene, 30 min cross-linked	10	308	30.3	2.87	10.6	[193]
6FDA-durene, 60 min cross-linked	10	308	2.14	0.40	5.35	[193]

*Permeability.

**Table 7:** Comparison between various technologies used for CO_2_ capture.

Technology	Advantages	Disadvantages	Scale
Absorption	(i) React rapidly (ii) High absorption capacities (iii) Very flexible	(i) Equipment corrosion (ii) High energy required for regenerating solvent	Industrial

Adsorption	(i) Low energy consumption and cost of CO_2_ capture (ii) Suitable for separating CO_2_ from dilute stream	Low adsorption capacities (in flue gases conditions)	Pilot

Cryogenic distillation	(i) Liquid CO_2_ production (ii) Not requiring solvents or other components (iii) Easy scaled-up to industrial-scale application	Require large amount of energy	Pilot

Membrane separation	(i) Clean and simple process (ii) Continuous, steady-state technology	Require high energy for post-combustion CO_2_ capture	Experimental

## References

[1] Solomon SQ, D SQ, Manning M (2009). Book reviews. *South African Geographical Journal*.

[2] McMillan CA, Keoleian GA, Spitzley DV (2005). *Greenhouse Gases*.

[3] Blasing TJ (2012). *Recent Greenhouse Gas Concentrations*.

[4] Chiao CH, Chen JL, Lan CR, Chen S, Hsu HW (2011). Development of carbon dioxide capture and storage technology taiwan power company perspective. *Sustainable Environment Research*.

[5] Dantas TLP, Luna FMT, Silva IJ (2011). Carbon dioxide-nitrogen separation through pressure swing adsorption. *Chemical Engineering Journal*.

[6] Zanganeh KE, Shafeen A, Salvador C (2009). CO_2_ capture and development of an advanced pilot-scale cryogenic separation and compression unit. *Energy Procedia*.

[7] A. T. A. o.C.a.N.A.C.o (2012). Canadian aviation industry report on greenhouse gas emissions reductions.

[8] Berstad D, Anantharaman R, Nekså P (2013). Low-temperature CO_2_capture technologies-applications and potential. *International Journal of Refrigeration*.

[9] Programme IEAIGGRD CO_2_ abatement in oil refineries: fired heaters.

[10] Zhao L, Riensche E, Menzer R, Blum L, Stolten D (2008). A parametric study of CO_2_/N_2_ gas separation membrane processes for post-combustion capture. *Journal of Membrane Science*.

[11] Hussain A, Hägg M-B (2010). A feasibility study of CO_2_ capture from flue gas by a facilitated transport membrane. *Journal of Membrane Science*.

[12] Forum IT Reducing transport greenhouse gas emissions: trends & data.

[13] Mahasenan N, Smith S, Humphreys K, Kaya Y The cement industry and global climate change: current and potential future cement industry CO_2_ emissions.

[14] Lackner KS, Park AHA, Miller BG (2010). Eliminating CO_2_ emissions from coal-fired power plants. *Generating Electricity in a Carbon-Constrained World*.

[15] Mohammadi A, Soltanieh M, Abbaspour M, Atabi F (2013). What is energy efficiency and emission reduction potential in the Iranian petrochemical industry?. *International Journal of Greenhouse Gas Control*.

[16] Worrell E, Price L, Martin N, Hendriks C, Meida LO (2001). Carbon dioxide emissions from the global cement industry. *Annual Review of Energy and the Environment*.

[17] Yang H, Xu Z, Fan M (2008). Progress in carbon dioxide separation and capture: a review. *Journal of Environmental Sciences*.

[18] Barnett J, Dessai S, Webber M (2004). Will OPEC lose from the Kyoto Protocol?. *Energy Policy*.

[19] Li H, Berrens RP, Bohara AK, Jenkins-Smith HC, Silva CL, Weimer DL (2004). Would developing country commitments affect US households’ support for a modified Kyoto Protocol?. *Ecological Economics*.

[20] Crombie M, Imbus S, Miracca I (2011). CO_2_ capture project phase 3-demonstration phase. *Energy Procedia*.

[21] Springer U (2003). The market for tradable GHG permits under the Kyoto Protocol: a survey of model studies. *Energy Economics*.

[22] Pridmore A, Bristow A, May T, Tight M (2003). Climate change, impacts, future scenarios and the role of transport. *Report of University of Leeds*.

[23] Olivier JGJ, Janssens-Maenhout G, Peters JAHW (2012). Trends in global CO_2_ emissions.

[24] Herzog H, Meldon J, Hatton A (2009). Advanced post-combustion CO_2_ capture.

[25] Pires JCM, Martins FG, Alvim-Ferraz MCM, Simões M (2011). Recent developments on carbon capture and storage: an overview. *Chemical Engineering Research and Design*.

[26] Chapel DG, Mariz CL, Ernest J Recovery of CO_2_ from flue gases: commercial trends.

[27] Zangeneh FT, Sahebdelfar S, Ravanchi MT (2011). Conversion of carbon dioxide to valuable petrochemicals: an approach to clean development mechanism. *Journal of Natural Gas Chemistry*.

[28] Dave N, Do T, Puxty G, Rowland R, Feron PHM, Attalla MI (2009). CO_2_ capture by aqueous amines and aqueous ammonia-A Comparison. *Energy Procedia*.

[29] Thiruvenkatachari R, Su S, An H, Yu XX (2009). Post combustion CO_2_ capture by carbon fibre monolithic adsorbents. *Progress in Energy and Combustion Science*.

[30] Gibbins J, Chalmers H (2008). Carbon capture and storage. *Energy Policy*.

[31] Metz B (2005). Carbon Dioxide Capture and Storage:. *Special Report of the Intergovernmental Panel on Climate Change*.

[32] Wall TF (2007). Combustion processes for carbon capture. *Proceedings of the Combustion Institute*.

[33] Rubin E, de Coninck H (2005). IPCC special report on carbon dioxide capture and storage.

[34] Choudhary VR, Mayadevi S, Singh AP (1995). Sorption isotherms of methane, ethane, ethene and carbon dioxide on NaX, NaY and Na-mordenite zeolites. *Journal of the Chemical Society, Faraday Transactions*.

[35] Dechamps P (2007). European CO_2_ capture and storage projects.

[36] Buhre BJP, Elliott LK, Sheng CD, Gupta RP, Wall TF (2005). Oxy-fuel combustion technology for coal-fired power generation. *Progress in Energy and Combustion Science*.

[37] Glazer M, Bertrand C, Fryda L, de Jong W (2010). EOSLT consortium biomass co-firing, WP 4—biomass co-firing in oxy-fuel combustion Part II: ash deposition modelling of coal and biomass blends under air and oxygen combustion conditions.

[38] SAGE Publications

[39] Olajire AA (2010). CO_2_ capture and separation technologies for end-of-pipe applications—a review. *Energy*.

[40] Samanta A, Zhao A, Shimizu GKH, Sarkar P, Gupta R (2012). Post-combustion CO_2_ capture using solid sorbents: a review. *Industrial and Engineering Chemistry Research*.

[41] Rhodes JS, Keith DW (2005). Engineering economic analysis of biomass IGCC with carbon capture and storage. *Biomass and Bioenergy*.

[42] Dantas TL, Rodrigues AE, Moreira RF Separation of carbon dioxide from flue gas using adsorption on porous solids.

[43] Esber III GS (2006). Carbon dioxide capture technology for the coal-powered electricity industry: a systematic prioritization of research needs.

[44] Lv Y, Yu X, Jia J, Tu S-T, Yan J, Dahlquist E (2012). Fabrication and characterization of superhydrophobic polypropylene hollow fiber membranes for carbon dioxide absorption. *Applied Energy*.

[45] Granite EJ, O’Brien T (2005). Review of novel methods for carbon dioxide separation from flue and fuel gases. *Fuel Processing Technology*.

[46] Nguyen T, Hilliard M, Rochelle GT (2010). Amine volatility in CO_2_ capture. *International Journal of Greenhouse Gas Control*.

[47] Gupta M, Coyle I, Thambimuthu K CO2capture technologies and opportunities in Canada.

[48] Herzog HJ (2000). The economics of CO_2_ separation and capture. *Journal of the Franklin Institute*.

[49] Pellegrini G, Strube R, Manfrida G (2010). Comparative study of chemical absorbents in postcombustion CO_2_ capture. *Energy*.

[50] MacDowell N, Florin N, Buchard A (2010). An overview of CO_2_ capture technologies. *Energy and Environmental Science*.

[51] Li Gang XP, Webley Paul A, Zhang Jun, Singh R Competition of CO_2_/H_2_O in adsorption based CO_2_ capture. *Energy Procedia*.

[52] Singh P, Niederer JPM, Versteeg GF (2007). Structure and activity relationships for amine based CO_2_ absorbents-I. *International Journal of Greenhouse Gas Control*.

[53] Ma'mun S (2005). Selection and characterization of new absorbents for carbon dioxide capture. *Chemical Engineering*.

[54] Cavenati S, Grande CA, Rodrigues AE (2006). Removal of carbon dioxide from natural gas by vacuum pressure swing adsorption. *Energy and Fuels*.

[55] David J (2000). *Economic evaluation of leading technology options 23 for sequestration of carbon dioxide [M.S. thesis]*.

[56] Albritton DL, Barker T, Bashmakov IA (2001). *Climate Change 2001: Synthesis Report*.

[57] Wang M, Lawal A, Stephenson P, Sidders J, Ramshaw C (2011). Post-combustion CO_2_ capture with chemical absorption: a state-of-the-art review. *Chemical Engineering Research and Design*.

[58] Gabrielsen J, Svendsen HF, Michelsen ML, Stenby EH, Kontogeorgis GM (2007). Experimental validation of a rate-based model for CO_2_ capture using an AMP solution. *Chemical Engineering Science*.

[59] Idem R, Wilson M, Tontiwachwuthikul P (2006). Pilot plant studies of the CO_2_ capture performance of aqueous MEA and mixed MEA/MDEA solvents at the University of Regina CO_2_ capture technology development plant and the boundary dam CO_2_ capture demonstration plant. *Industrial and Engineering Chemistry Research*.

[60] Lucquiaud M, Gibbins J (2011). On the integration of CO_2_ capture with coal-fired power plants: a methodology to assess and optimise solvent-based post-combustion capture systems. *Chemical Engineering Research and Design*.

[61] Knudsen JN, Jensen JN, Vilhelmsen PJ, Biede O (2009). Experience with CO_2_ capture from coal flue gas in pilot-scale: testing of different amine solvents. *Energy Procedia*.

[62] Feron PHM (2010). Exploring the potential for improvement of the energy performance of coal fired power plants with post-combustion capture of carbon dioxide. *International Journal of Greenhouse Gas Control*.

[63] Qin F, Wang S, Hartono A, Svendsen HF, Chen C (2010). Kinetics of CO_2_ absorption in aqueous ammonia solution. *International Journal of Greenhouse Gas Control*.

[64] Mangalapally HP, Notz R, Hoch S (2009). Pilot plant experimental studies of post combustion CO_2_ capture by reactive absorption with MEA and new solvents. *Energy Procedia*.

[65] Kumar PS, Hogendoorn JA, Versteeg GF, Feron PHM (2003). Kinetics of the reaction of *CO_2_* with aqueous potassium salt of taurine and glycine. *AIChE Journal*.

[66] Freeman SA, Dugas R, van Wagener D, Nguyen T, Rochelle GT (2009). Carbon dioxide capture with concentrated, aqueous piperazine. *Energy Procedia*.

[67] Holst JV, Versteeg GF, Brilman DWF, Hogendoorn JA (2009). Kinetic study of CO_2_ with various amino acid salts in aqueous solution. *Chemical Engineering Science*.

[68] Hamborg ES, Niederer JPM, Versteeg GF (2007). Dissociation constants and thermodynamic properties of amino acids used in CO_2_ absorption from (293 to 353) K. *Journal of Chemical and Engineering Data*.

[69] Aronu UE, Svendsen HF, Hoff KA (2010). Investigation of amine amino acid salts for carbon dioxide absorption. *International Journal of Greenhouse Gas Control*.

[70] Yeh JT, Resnik KP, Rygle K, Pennline HW (2005). Semi-batch absorption and regeneration studies for CO_2_ capture by aqueous ammonia. *Fuel Processing Technology*.

[71] Yu CH, Huang CH, Tan CS (2012). A Review of CO_2_ Capture by Absorption and Adsorption. *Aerosol and Air Quality Research*.

[72] Gurkan BE, Juan C, Mindrup EM Chemically complexing ionic liquids for post-combustion CO_2_ capture.

[73] Bates ED, Mayton RD, Ntai I, Davis JH (2002). CO_2_ capture by a task-specific ionic liquid. *Journal of the American Chemical Society*.

[74] Baj S, Siewniak A, Chrobok A, Krawczyk T, Sobolewski A (2012). Monoethanolamine and ionic liquid aqueous solutions as effective systems for CO_2_capture. *Journal of Chemical Technology and Biotechnology*.

[75] Ciferno JP, Lang D, Rochelle GT (2010). *Carbon Dioxide Capture by Absorption with Potassium Carbonate*.

[76] Cullinane JT, Rochelle GT (2005). Thermodynamics of aqueous potassium carbonate, piperazine, and carbon dioxide. *Fluid Phase Equilibria*.

[77] Mangalapally HP, Hasse H (2011). Pilot plant experiments with mea and new solvents for post combustion CO_2_ capture by reactive absorption. *Energy Procedia*.

[78] Brouwer J, Feron P, Ten Asbroek N Amino-acid salts for CO_2_ capture from flue gases.

[79] Kang D, Park S, Jo H, Min J, Park J (2013). Solubility of CO_2_in amino-acid-based solutions of (potassium sarcosinate), (potassium alaninate + piperazine), and (potassium serinate + piperazine). *Journal of Chemical & Engineering Data*.

[80] Farid B, Fadwa E Front matter.

[81] Davidson RM (2007). *Post-Combustion Carbon Capture from Coal Fired Plants: Solvent Scrubbing*.

[82] Darde V, Thomsen K, van Well WJ, Stenby EH (2009). Chilled ammonia process for CO_2_ capture. *Energy Procedia*.

[83] Bishnoi S, Rochelle GT (2002). Thermodynamics of piperazine/methyldiethanolamine/water/carbon dioxide. *Industrial and Engineering Chemistry Research*.

[84] Bajpai A, Mondal MK (2013). Equilibrium solubility of CO_2_in aqueous mixtures of DEA and AEEA. *Journal of Chemical & Engineering Data *.

[85] Chaffee AL, Knowles GP, Liang Z, Zhang J, Xiao P, Webley PA (2007). CO_2_ capture by adsorption: materials and process development. *International Journal of Greenhouse Gas Control*.

[86] Li J-R, Ma Y, McCarthy MC (2011). Carbon dioxide capture-related gas adsorption and separation in metal-organic frameworks. *Coordination Chemistry Reviews*.

[87] Meng L-Y, Park S-J (2012). Influence of MgO template on carbon dioxide adsorption of cation exchange resin-based nanoporous carbon. *Journal of Colloid and Interface Science*.

[88] Sevilla M, Fuertes AB (2012). CO_2_ adsorption by activated templated carbons. *Journal of Colloid and Interface Science*.

[89] Martunus M, Helwani Z, Wiheeb AD, Kim J, Othman MR (2012). Improved carbon dioxide capture using metal reinforced hydrotalcite under wet conditions. *International Journal of Greenhouse Gas Control*.

[90] Dou B, Song Y, Liu Y, Feng C (2010). High temperature CO_2_ capture using calcium oxide sorbent in a fixed-bed reactor. *Journal of Hazardous Materials*.

[91] Kotyczka-moranska M, Tomaszewicz G, Labojko G (2012). Comparison of different methods for enhancing CO_2_capture by CaO-based sorbents. Review. *Physicochemical Problems of Mineral Processing*.

[92] Valenti G, Bonalumi D, Macchi E (2011). A parametric investigation of the chilled ammonia process from energy and economic perspectives. *Fuel*.

[93] Lee ZH, Lee KT, Bhatia S, Mohamed AR (2012). Post-combustion carbon dioxide capture: evolution towards utilization of nanomaterials. *Renewable and Sustainable Energy Reviews*.

[94] Xiang Z, Hu Z, Cao D (2011). Metal-organic frameworks with incorporated carbon nanotubes: improving carbon dioxide and methane storage capacities by lithium doping. *Angewandte Chemie*.

[95] Essaki K, Kato M, Nakagawa K (2006). CO_2_ removal at high temperature using packed bed of lithium silicate pellets. *Journal of the Ceramic Society of Japan*.

[96] Martavaltzi CS, Lemonidou AA (2008). Development of new CaO based sorbent materials for CO_2_ removal at high temperature. *Microporous and Mesoporous Materials*.

[97] Besson R, Rocha Vargas M, Favergeon L (2012). CO_2_ adsorption on calcium oxide: an atomic-scale simulation study. *Surface Science*.

[98] Miyata S (1983). Anion-exchange properties of hydrotalcite-like compounds. *Clays & Clay Minerals*.

[99] Mohamed AR, Bhatia S, Lee KT, Foo CYH, Lee ZH, Razali NA (2012). Nanomaterials as environmentally compatible next generation green carbon capture and utilization materials. *Transactions on GIGAKU*.

[100] Songolzadeh M, Takht Ravanchi M, Soleimani M (2012). Carbon dioxide capture and storage: a general review on adsorbents. *World Academy of Science, Engineering and Technology*.

[101] Anbia M, Hoseini V (2012). Development of MWCNT@MIL-101 hybrid composite with enhanced adsorption capacity for carbon dioxide. *Chemical Engineering Journal*.

[102] Lin L-Y, Bai H (2010). Continuous generation of mesoporous silica particles via the use of sodium metasilicate precursor and their potential for CO_2_ capture. *Microporous and Mesoporous Materials*.

[103] D’Alessandro DM, Smit B, Long JR (2010). Carbon dioxide capture: prospects for new materials. *Angewandte Chemie*.

[104] Jang DI, Park SJ (2012). Influence of nickel oxide on carbon dioxide adsorption behaviors of activated carbons. *Fuel*.

[105] Choi S, Drese JH, Jones CW (2009). Adsorbent materials for carbon dioxide capture from large anthropogenic point sources. *ChemSusChem*.

[106] Delgado JA, Uguina MA, Sotelo JL, Ruíz B (2006). Fixed-bed adsorption of carbon dioxide-helium, nitrogen-helium and carbon dioxide-nitrogen mixtures onto silicalite pellets. *Separation and Purification Technology*.

[107] Abid HR, Pham GH, Ang H-M, Tade MO, Wang S (2012). Adsorption of CH_4_ and CO_2_ on Zr-metal organic frameworks. *Journal of Colloid and Interface Science*.

[108] Wang J, Stevens LA, Drage TC, Wood J (2012). Preparation and CO_2_ adsorption of amine modified Mg-Al LDH via exfoliation route. *Chemical Engineering Science*.

[109] Mishra AK, Ramaprabhu S (2012). Palladium nanoparticles decorated graphite nanoplatelets for room temperature carbon dioxide adsorption. *Chemical Engineering Journal*.

[110] Finos G, Collins S, Blanco G (2012). Infrared spectroscopic study of carbon dioxide adsorption on the surface of cerium-gallium mixed oxides. *Catalysis Today*.

[111] Grimm RP, Eriksson KA, Ripepi N, Eble C, Greb SF (2012). Seal evaluation and confinement screening criteria for beneficial carbon dioxide storage with enhanced coal bed methane recovery in the Pocahontas Basin, Virginia. *International Journal of Coal Geology*.

[112] Guo B, Chang L, Xie K (2006). Adsorption of carbon dioxide on activated carbon. *Journal of Natural Gas Chemistry*.

[113] Sakurovs R, Day S, Weir S (2012). Relationships between the sorption behaviour of methane, carbon dioxide, nitrogen and ethane on coals. *Fuel*.

[114] Weniger P, Franců J, Hemza P, Krooss BM (2012). Investigations on the methane and carbon dioxide sorption capacity of coals from the SW Upper Silesian Coal Basin, Czech Republic. *International Journal of Coal Geology*.

[115] Garnier C, Finqueneisel G, Zimny T (2011). Selection of coals of different maturities for CO_2_ Storage by modelling of CH_4_ and CO_2_ adsorption isotherms. *International Journal of Coal Geology*.

[116] Abanades JC, Rubin ES, Anthony EJ (2004). Sorbent cost and performance in CO_2_ capture systems. *Industrial and Engineering Chemistry Research*.

[117] Drage TC, Blackman JM, Pevida C, Snape CE (2009). Evaluation of activated carbon adsorbents for CO_2_ capture in gasification. *Energy and Fuels*.

[118] Shen W, Zhang S, He Y, Li J, Fan W (2011). Hierarchical porous polyacrylonitrile-based activated carbon fibers for CO_2_ capture. *Journal of Materials Chemistry*.

[119] Gray M, Soong Y, Champagne K, Stevens R, Toochinda P, Chuang S (2001). Solid amine CO2capture sorbents. *Fuel*.

[120] Pevida C, Plaza MG, Arias B, Fermoso J, Rubiera F, Pis JJ (2008). Surface modification of activated carbons for CO_2_ capture. *Applied Surface Science*.

[121] Plaza MG, Pevida C, Arias B, Fermoso J, Rubiera F, Pis JJ (2009). A comparison of two methods for producing CO_2_ capture adsorbents. *Energy Procedia*.

[122] Plaza MG, García S, Rubiera F, Pis JJ, Pevida C (2010). Post-combustion CO_2_ capture with a commercial activated carbon: comparison of different regeneration strategies. *Chemical Engineering Journal*.

[123] Nor Kamarudin KS, Mat H (2009). Synthesis and modification of micro and mesoporous materials as CO_2_ adsorbent.

[124] Radosz M, Hu X, Krutkramelis K, Shen Y (2008). Flue-gas carbon capture on carbonaceous sorbents: toward a low-cost multifunctional carbon filter for “green” energy producers. *Industrial and Engineering Chemistry Research*.

[125] Rosas JM, Bedia J, Rodríguez-Mirasol J, Cordero T (2008). Preparation of hemp-derived activated carbon monoliths. Adsorption of water vapor. *Industrial and Engineering Chemistry Research*.

[126] Yang R, Liu G, Li M, Zhang J, Hao X (2012). Preparation and N2, CO_2_ and H_2_ adsorption of super activated carbon derived from biomass source hemp (Cannabis sativa L.) stem. *Microporous and Mesoporous Materials*.

[127] Siriwardane RV, Shen M-S, Fisher EP, Poston JA (2001). Adsorption of CO_2_ on molecular sieves and activated carbon. *Energy and Fuels*.

[128] Vatalis KI, Laaksonen A, Charalampides G, Benetis NP (2012). Intermediate technologies towards low-carbon economy. the Greek zeolite CCS outlook into the EU commitments. *Renewable and Sustainable Energy Reviews*.

[129] Liu Z, Grande CA, Li P, Yu J, Rodrigues AE (2011). Multi-bed vacuum pressure swing adsorption for carbon dioxide capture from flue gas. *Separation and Purification Technology*.

[130] Zhang J, Xiao P, Li G, Webley PA (2009). Effect of flue gas impurities on CO_2_ capture performance from flue gas at coal-fired power stations by vacuum swing adsorption. *Energy Procedia*.

[131] Cui X, Bustin RM, Dipple G (2004). Selective transport of CO_2_, CH_4_, and N_2_ in coals: insights from modeling of experimental gas adsorption data. *Fuel*.

[132] Anderson CJ, Tao W, Jiang J, Sandler SI, Stevens GW, Kentish SE (2011). An experimental evaluation and molecular simulation of high temperature gas adsorption on nanoporous carbon. *Carbon*.

[133] Kumar M, Ando Y (2010). Chemical vapor deposition of carbon nanotubes: a review on growth mechanism and mass production. *Journal of Nanoscience and Nanotechnology*.

[134] Cinke M, Li J, Bauschlicher CW, Ricca A, Meyyappan M (2003). CO_2_ adsorption in single-walled carbon nanotubes. *Chemical Physics Letters*.

[135] Portugal AF, Derks PWJ, Versteeg GF, Magalhães FD, Mendes A (2007). Characterization of potassium glycinate for carbon dioxide absorption purposes. *Chemical Engineering Science*.

[136] Banerjee R, Phan A, Wang B (2008). High-throughput synthesis of zeolitic imidazolate frameworks and application to CO_2_ capture. *Science*.

[137] Park KS, Ni Z, Côté AP (2006). Exceptional chemical and thermal stability of zeolitic imidazolate frameworks. *Proceedings of the National Academy of Sciences of the United States of America*.

[138] Burchell TD, Judkins RR (1996). Passive CO_2_ removal using a carbon fiber composite molecular sieve. *Energy Conversion and Management*.

[139] Yong Z, Mata V, Rodrigues AE (2002). Adsorption of carbon dioxide at high temperature—a review. *Separation and Purification Technology*.

[140] Kimber GM, Jagtoyen M, Fei YQ, Derbyshire FJ (1996). Fabrication of carbon fibre composites for gas separation. *Gas Separation and Purification*.

[141] Viculis LM, Mack JJ, Mayer OM, Hahn HT, Kaner RB (2005). Intercalation and exfoliation routes to graphite nanoplatelets. *Journal of Materials Chemistry*.

[142] Mishra AK, Ramaprabhu S (2010). Study of CO_2_ adsorption in low cost graphite nanoplatelets. *International Journal of Chemical Engineering and Applications*.

[143] Du R, Feng X, Chakma A (2006). Poly(N,N-dimethylaminoethyl methacrylate)/polysulfone composite membranes for gas separations. *Journal of Membrane Science*.

[144] Kumar K, Dasgupta CN, Nayak B, Lindblad P, Das D (2011). Development of suitable photobioreactors for CO_2_ sequestration addressing global warming using green algae and cyanobacteria. *Bioresource Technology*.

[145] Deng H, Yi H, Tang X, Yu Q, Ning P, Yang L (2012). Adsorption equilibrium for sulfur dioxide, nitric oxide, carbon dioxide, nitrogen on 13X and 5A zeolites. *Chemical Engineering Journal*.

[146] Gargiulo N, Pepe F, Caputo D (2012). Modeling carbon dioxide adsorption on polyethylenimine-functionalized TUD-1 mesoporous silica. *Journal of Colloid and Interface Science*.

[147] Millward AR, Yaghi OM (2005). Metal-organic frameworks with exceptionally high capacity for storage of carbon dioxide at room temperature. *Journal of the American Chemical Society*.

[148] Lu C, Bai H, Su F, Chen W, Hwang JF, Lee H-H (2010). Adsorption of carbon dioxide from gas streams via mesoporous spherical-silica particles. *Journal of the Air and Waste Management Association*.

[149] Boonpoke A, Chiarakorn S, Laosiripojana N, Towprayoon S, Chidthaisong A (2011). synthesis of activated carbon and MCM-41 from bagasse and rice husk and their carbon dioxide adsorption capacity. *Journal of Sustainable Energy & Environmentn*.

[150] Wei J, Liao L, Xiao Y, Zhang P, Shi Y (2010). Capture of carbon dioxide by amine-impregnated as-synthesized MCM-41. *Journal of Environmental Sciences*.

[151] Wang Q, Tay HH, Zhong Z, Luo J, Borgna A (2012). Synthesis of high-temperature CO_2_adsorbents from organo-layered double hydroxides with markedly improved CO_2_capture capacity. *Energy & Environmental Science*.

[152] Lin H, Freeman BD (2004). Gas solubility, diffusivity and permeability in poly(ethylene oxide). *Journal of Membrane Science*.

[153] Chowdhury P, Bikkina C, Gumma S (2009). Gas adsorption properties of the chromium-based metal organic framework MIL-101. *The Journal of Physical Chemistry C*.

[154] Li P, Ge B, Zhang S, Chen S, Zhang Q, Zhao Y (2008). CO_2_ capture by polyethylenimine-modified fibrous adsorbent. *Langmuir*.

[155] Aziz B, Hedin N, Bacsik Z (2012). Quantification of chemisorption and physisorption of carbon dioxide on porous silica modified by propylamines: effect of amine density. *Microporous and Mesoporous Materials*.

[156] Maroto-Valer MM, Tang Z, Zhang Y (2005). CO_2_ capture by activated and impregnated anthracites. *Fuel Processing Technology*.

[157] Xu X, Song C, Miller BG, Scaroni AW (2005). Adsorption separation of carbon dioxide from flue gas of natural gas-fired boiler by a novel nanoporous “molecular basket” adsorbent. *Fuel Processing Technology*.

[158] Xu X, Song C, Andresen JM, Miller BG, Scaroni AW (2002). Novel polyethylenimine-modified mesoporous molecular sieve of MCM-41 type as high-capacity adsorbent for CO_2_ capture. *Energy and Fuels*.

[159] Zhang J, Singh R, Webley PA (2008). Alkali and alkaline-earth cation exchanged chabazite zeolites for adsorption based CO_2_ capture. *Microporous and Mesoporous Materials*.

[160] Abid HR, Tian H, Ang H-M, Tade MO, Buckley CE, Wang S (2012). Nanosize Zr-metal organic framework (UiO-66) for hydrogen and carbon dioxide storage. *Chemical Engineering Journal*.

[161] Chen C, Kim J, Ahn W-S (2012). Efficient carbon dioxide capture over a nitrogen-rich carbon having a hierarchical micro-mesopore structure. *Fuel*.

[162] Plaza MG, Pevida C, Arias B (2008). Application of thermogravimetric analysis to the evaluation of aminated solid sorbents for CO_2_ capture. *Journal of Thermal Analysis and Calorimetry*.

[163] Park A-Y, Kwon H, Woo AJ, Kim S-J (2005). Layered double hydroxide surface modified with (3-aminopropyl) triethoxysilane by covalent bonding. *Advanced Materials*.

[164] Nhlapo NS (2008). Intercalation of fatty acids into layered double hydroxides.

[165] Shafeeyan MS, Wan Daud WMA, Houshmand A, Arami-Niya A (2012). The application of response surface methodology to optimize the amination of activated carbon for the preparation of carbon dioxide adsorbents. *Fuel*.

[166] Clausse M, Merel J, Meunier F (2011). Numerical parametric study on CO_2_ capture by indirect thermal swing adsorption. *International Journal of Greenhouse Gas Control*.

[167] Wang L, Liu Z, Li P, Yu J, Rodrigues AE (2012). Experimental and modeling investigation on post-combustion carbon dioxide capture using zeolite 13X-APG by hybrid VTSA process. *Chemical Engineering Journal*.

[168] Kulkarni AR, Sholl DS (2012). Analysis of Equilibrium-Based TSA Processes for Direct Capture of CO_2_from Air. *Industrial & Engineering Chemistry Research*.

[169] Merel J, Clausse M, Meunier F (2008). Experimental investigation on CO_2_ post-combustion capture by indirect thermal swing adsorption using 13X and 5A zeolites. *Industrial and Engineering Chemistry Research*.

[170] Lucas S, Calvo MP, Palencia C, Cocero MJ (2004). Mathematical model of supercritical CO_2_ adsorption on activated carbon: effect of operating conditions and adsorption scale-up. *Journal of Supercritical Fluids*.

[171] Hoeger C, Bence C, Burt SS, Baxter A, Baxter L Cryogenic CO_2_ capture for improved efficiency at reduced cost.

[172] Burt S, Baxter A, Baxter L Cryogenic CO_2_ capture to control climate change emissions.

[173] Tuinier MJ, Hamers HP, van Sint Annaland M (2011). Techno-economic evaluation of cryogenic CO_2_ capture-A comparison with absorption and membrane technology. *International Journal of Greenhouse Gas Control*.

[174] Hart A, Gnanendran N (2009). Cryogenic CO_2_ capture in natural gas. *Energy Procedia*.

[175] Xu G, Li L, Yang Y, Tian L, Liu T, Zhang K (2012). A novel CO_2_ cryogenic liquefaction and separation system. *Energy*.

[176] Shimekit B, Mukhtar H, Hamid AM (2012). Natural gas purification technologies-major advances for CO_2_ separation and future directions. *Advances in Natural Gas Technology*.

[177] Ravanchi MT, Sahebdelfar S, Zangeneh FT (2011). Carbon dioxide sequestration in petrochemical industries with the aim of reduction in greenhouse gas emissions. *Frontiers of Chemical Engineering in China*.

[178] Lively RP, Koros WJ, Johnson JR (2012). Enhanced cryogenic CO_2_ capture using dynamically operated low-cost fiber beds. *Chemical Engineering Science*.

[179] Clodic D, El Hitti R, Younes M, Bill A, Casier F CO_2_capture by anti-sublimation thermo-economic process evaluation.

[180] Amann J-M, Kanniche M, Bouallou C (2009). Natural gas combined cycle power plant modified into an O_2_/CO_2_ cycle for CO_2_ capture. *Energy Conversion and Management*.

[181] Song C-F, Kitamura Y, Li S-H, Ogasawara K (2012). Design of a cryogenic CO_2_ capture system based on Stirling coolers. *International Journal of Greenhouse Gas Control*.

[182] Tuinier MJ, van Sint Annaland M, Kuipers JAM (2011). A novel process for cryogenic CO_2_ capture using dynamically operated packed beds-An experimental and numerical study. *International Journal of Greenhouse Gas Control*.

[183] Chiesa P, Campanari S, Manzolini G (2011). CO_2_ cryogenic separation from combined cycles integrated with molten carbonate fuel cells. *International Journal of Hydrogen Energy*.

[184] Favre E (2011). Membrane processes and postcombustion carbon dioxide capture: challenges and prospects. *Chemical Engineering Journal*.

[185] Freeman B, Yampolskii Y (2010). *Membrane Gas Separation*.

[186] Bounaceur R, Lape N, Roizard D, Vallieres C, Favre E (2006). Membrane processes for post-combustion carbon dioxide capture: a parametric study. *Energy*.

[187] Aaron D, Tsouris C (2005). Separation of CO_2_ from flue gas: a review. *Separation Science and Technology*.

[188] Xu A, Yang A, Young S, deMontigny D, Tontiwachwuthikul P (2008). Effect of internal coagulant on effectiveness of polyvinylidene fluoride membrane for carbon dioxide separation and absorption. *Journal of Membrane Science*.

[189] Chew T-L, Ahmad AL, Bhatia S (2010). Ordered mesoporous silica (OMS) as an adsorbent and membrane for separation of carbon dioxide (CO_2_). *Advances in Colloid and Interface Science*.

[190] Scholes CA, Chen GQ, Stevens GW, Kentish SE (2011). Nitric oxide and carbon monoxide permeation through glassy polymeric membranes for carbon dioxide separation. *Chemical Engineering Research and Design*.

[191] Scholes CA, Kentish SE, Stevens GW (2008). Carbon dioxide separation through polymeric membrane systems for flue gas applications. *Recent Patents on Chemical Engineering*.

[192] El-Azzami LA, Grulke EA (2008). Carbon dioxide separation from hydrogen and nitrogen by fixed facilitated transport in swollen chitosan membranes. *Journal of Membrane Science*.

[193] Powell CE, Qiao GG (2006). Polymeric CO_2_/N_2_ gas separation membranes for the capture of carbon dioxide from power plant flue gases. *Journal of Membrane Science*.

[194] El-Azzami LA, Grulke EA (2009). Carbon dioxide separation from hydrogen and nitrogen: facilitated transport in arginine salt-chitosan membranes. *Journal of Membrane Science*.

[195] Xomeritakis G, Tsai C-Y, Brinker CJ (2005). Microporous sol-gel derived aminosilicate membrane for enhanced carbon dioxide separation. *Separation and Purification Technology*.

[196] Favre E (2007). Carbon dioxide recovery from post-combustion processes: can gas permeation membranes compete with absorption?. *Journal of Membrane Science*.

[197] Julbe A (2007). Chapter 6 Zeolite membranes—synthesis, characterization and application. *Studies in Surface Science and Catalysis*.

[198] Shin DW, Hyun SH, Cho CH, Han MH (2005). Synthesis and CO_2_/N_2_ gas permeation characteristics of ZSM-5 zeolite membranes. *Microporous and Mesoporous Materials*.

[199] Anderson M, Lin YS (2010). Carbonate-ceramic dual-phase membrane for carbon dioxide separation. *Journal of Membrane Science*.

[200] Shekhawat D, Luebke DR, Pennline HW (2003). A review of carbon dioxide selective membranes. *A Topical Report*.

[201] Kumar P, Kim S, Ida J, Guliants VV (2008). Polyethyleneimine-modified MCM-48 membranes: effect of water vapor and feed concentration on N_2_/CO_2_ selectivity. *Industrial and Engineering Chemistry Research*.

[202] Merkel TC, Lin H, Wei X, Baker R (2010). Power plant post-combustion carbon dioxide capture: an opportunity for membranes. *Journal of Membrane Science*.

[203] Cai Y, Wang Z, Yi C, Bai Y, Wang J, Wang S (2008). Gas transport property of polyallylamine-poly(vinyl alcohol)/polysulfone composite membranes. *Journal of Membrane Science*.

[204] Deng L, Kim T-J, Hägg M-B (2009). Facilitated transport of CO_2_ in novel PVAm/PVA blend membrane. *Journal of Membrane Science*.

[205] Ren X, Ren J, Li H, Feng S, Deng M (2012). Poly (amide-6-b-ethylene oxide) multilayer composite membrane for carbon dioxide separation. *International Journal of Greenhouse Gas Control*.

[206] Liu L, Chakma A, Feng X (2004). Preparation of hollow fiber poly(ether block amide)/polysulfone composite membranes for separation of carbon dioxide from nitrogen. *Chemical Engineering Journal*.

[207] Car A, Stropnik C, Yave W, Peinemann K-V (2008). PEG modified poly(amide-b-ethylene oxide) membranes for CO_2_ separation. *Journal of Membrane Science*.

[208] Yave W, Car A, Peinemann K-V (2010). Nanostructured membrane material designed for carbon dioxide separation. *Journal of Membrane Science*.

[209] Gu Y, Oyama ST (2007). High molecular permeance in a poreless ceramic membrane. *Advanced Materials*.

[210] Reif M, Dittmeyer R (2003). Porous, catalytically active ceramic membranes for gas-liquid reactions: a comparison between catalytic diffuser and forced through flow concept. *Catalysis Today*.

[211] Kusakabe K, Kuroda T, Morooka S (1998). Separation of carbon dioxide from nitrogen using ion-exchanged faujasite-type zeolite membranes formed on porous support tubes. *Journal of Membrane Science*.

[212] van den Bergh J, Zhu W, Gascon J, Moulijn JA, Kapteijn F (2008). Separation and permeation characteristics of a DD3R zeolite membrane. *Journal of Membrane Science*.

[213] Bernal MP, Coronas J, Menéndez M, Santamaría J (2004). Separation of CO_2_/N_2_ mixtures using MFI-type zeolite membranes. *AIChE Journal*.

[214] Rui Z, Ji H, Lin YS (2011). Modeling and analysis of ceramic-carbonate dual-phase membrane reactor for carbon dioxide reforming with methane. *International Journal of Hydrogen Energy*.

[215] Rui Z, Anderson M, Lin YS, Li Y (2009). Modeling and analysis of carbon dioxide permeation through ceramic-carbonate dual-phase membranes. *Journal of Membrane Science*.

[216] Metz SJ, Mulder MHV, Wessling M (2004). Gas-permeation properties of poly(ethylene oxide) poly(butylene terephthalate) block copolymers. *Macromolecules*.

[217] Xu Z, Wang J, Chen W, Xu Y Separation and fixation of carbon dioxide using polymeric membrane contactor.

[218] Dortmundt D, Doshi K (1999). *Recent Developments in CO_2_ Removal Membrane Technology*.

[219] Scholes CA, Kentish SE, Stevens GW (2009). The effect of condensable minor components on the gas separation performance of polymeric membranes for carbon dioxide capture. *Energy Procedia*.

[220] Hunger K, Schmeling N, Jeazet HB, Janiak C, Staudt C, Kleinermanns K (2012). Investigation of cross-linked and additive containing polymer materials for membranes with improved performance in pervaporation and gas separation. *Membrane*.

[221] Reijerkerk SR (2010). Polyether based block copolymer membranes for CO_2_ separation. *Science and Technology*.

[222] Ahmad ALB, Jawad ZA, Low SC, Zein HS (2012). Prospect of mixed matrix membrane towards CO_2_Separation. *Journal of Membrane Science & Technology*.

[223] Scholes CA, Chen GQ, Stevens GW, Kentish SE (2010). Plasticization of ultra-thin polysulfone membranes by carbon dioxide. *Journal of Membrane Science*.

[224] Du N, Park HB, Robertson GP (2011). Polymer nanosieve membranes for CO_2_-capture applications. *Nature Materials*.

[225] Uchytil P, Schauer J, Petrychkovych R, Setnickova K, Suen SY (2011). Ionic liquid membranes for carbon dioxide-methane separation. *Journal of Membrane Science*.

[226] Nik OG, Chen XY, Kaliaguine S (2011). Amine-functionalized zeolite FAU/EMT-polyimide mixed matrix membranes for CO_2_/CH_4_ separation. *Journal of Membrane Science*.

[227] Hudiono YC, Carlisle TK, Bara JE, Zhang Y, Gin DL, Noble RD (2010). A three-component mixed-matrix membrane with enhanced CO_2_ separation properties based on zeolites and ionic liquid materials. *Journal of Membrane Science*.

[228] Kovvali A, Obuskovic G Immobilized liquid membranes for CO_2_ separation'.

[229] Wang Z, Achenie LEK, Khativ SJ, Oyama ST (2012). Simulation study of single-gas permeation of carbon dioxide and methane in hybrid inorganic-organic membrane. *Journal of Membrane Science*.

[230] Yan S-P, Fang M-X, Zhang W-F (2007). Experimental study on the separation of CO_2_ from flue gas using hollow fiber membrane contactors without wetting. *Fuel Processing Technology*.

[231] Li J-L, Chen B-H (2005). Review of CO_2_ absorption using chemical solvents in hollow fiber membrane contactors. *Separation and Purification Technology*.

[232] Kim Y-S, Yang S-M (2000). Absorption of carbon dioxide through hollow fiber membranes using various aqueous absorbents. *Separation and Purification Technology*.

[233] Sugiura K, Takei K, Tanimoto K, Miyazaki Y (2003). The carbon dioxide concentrator by using MCFC. *Journal of Power Sources*.

[234] Herzog H (2003). Assessing the feasibility of capturing CO_2_ from the air.

[235] Abu-Zahra MRM, Niederer JPM, Feron PHM, Versteeg GF (2007). CO_2_ capture from power plants. Part II. A parametric study of the economical performance based on mono-ethanolamine. *International Journal of Greenhouse Gas Control*.

